# Supplementary figures and images for: Autophagy-related protein Atg11 is essential for microtubule-mediated chromosome segregation
Source: PLoS Biol. 2025 Apr 2;23(4):e3003069. doi: 10.1371/journal.pbio.3003069 (PMC11984983; doi:10.1371/journal.pbio.3003069)

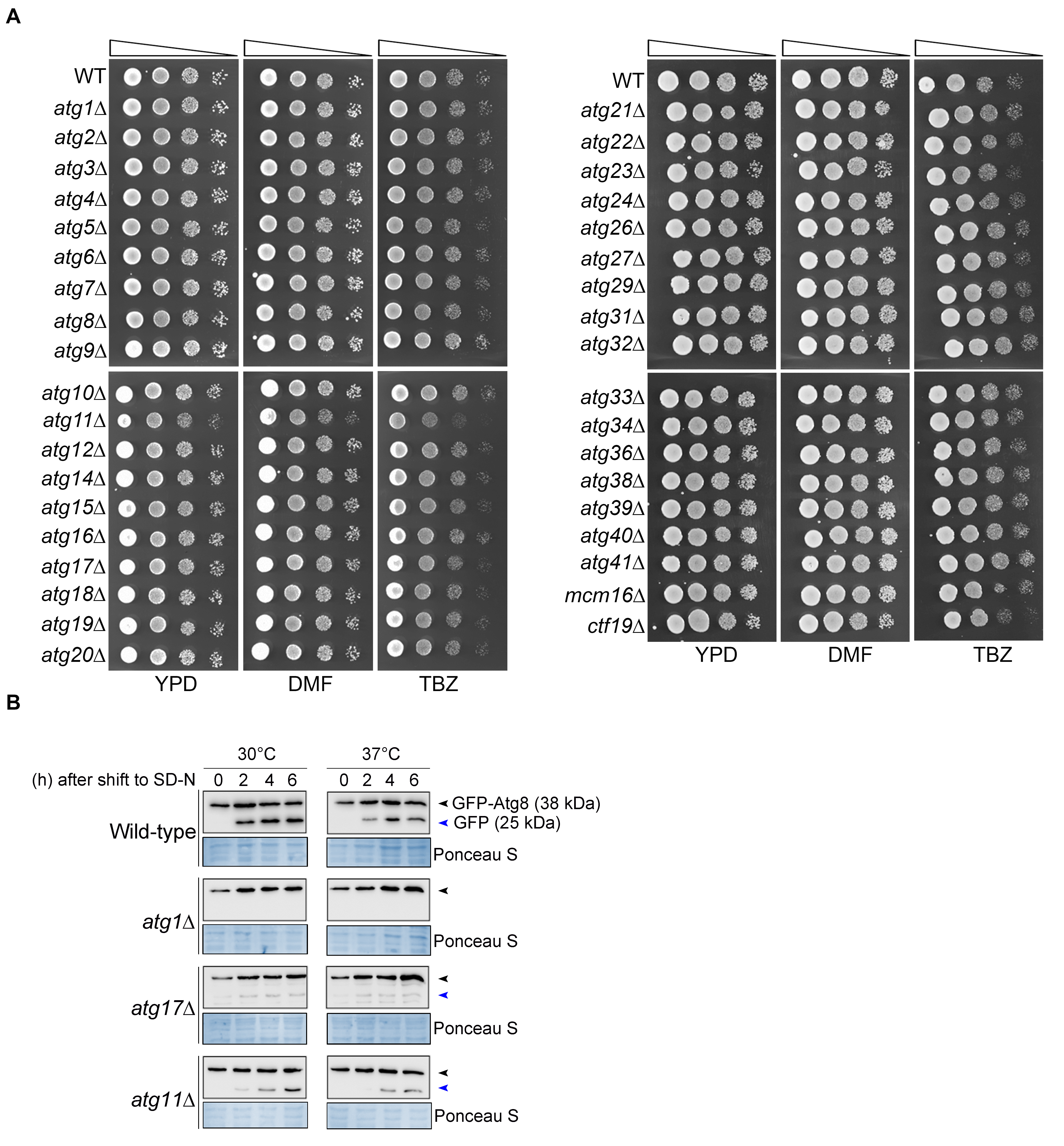

Supplement: S1 Fig — (A) Overnight grown cells of wild-type, null mutants of autophagy-related genes (atg), and two kinetochore mutants, mcm16∆ and ctf19∆ , were 10-fold serially diluted, spotted on YPD and YPD plates containing dimethylformamide (DMF) only or 50 μg mL−1 thiabendazole (TBZ). Plates were photographed after incubation at 30 °C for 36 h. (B) Immunoblot analysis of whole-cell lysate prepared at indicated time points from the wild-type, atg1∆ , atg17∆ , and atg11∆ strains expressing GFP-Atg8 grown at 30 and 37 °C in nitrogen starvation medium and probed with anti-GFP antibodies. GFP-Atg8 (38 kDa) and free GFP (25 kDa) are labeled with black and blue arrowheads, respectively. Uncropped western blots are available in S1 Raw Images. (TIF) [file pbio.3003069.s001.tif]

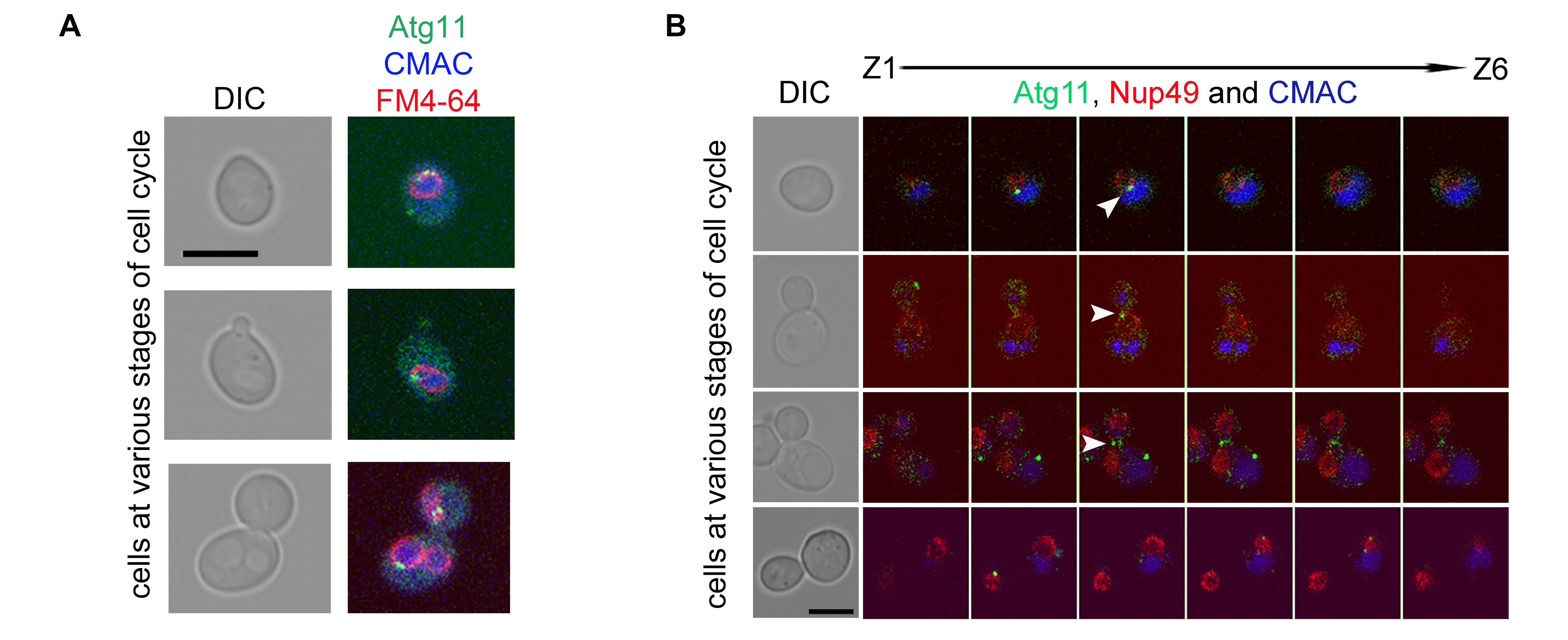

Supplement: S2 Fig — (A) Micrographs showing canonical localization of Atg11 onto the vacuolar membrane during mitosis. The sfGFP-Atg11 expressing cells were co-stained with FM4–64 (staining vacuolar membrane, red) and with CMAC (staining the vacuolar lumen, blue). Scale bar, 5 µm. (B) Micrographs representing different focal planes showing localization of Atg11 (sfGFP-Atg11) closer to the nuclear envelope (Nup49-mCherry, white arrowheads), at various cell cycle stages and co-stained with the vacuolar lumen dye CMAC. Scale bar, 6 µm. (TIF) [file pbio.3003069.s002.tif]

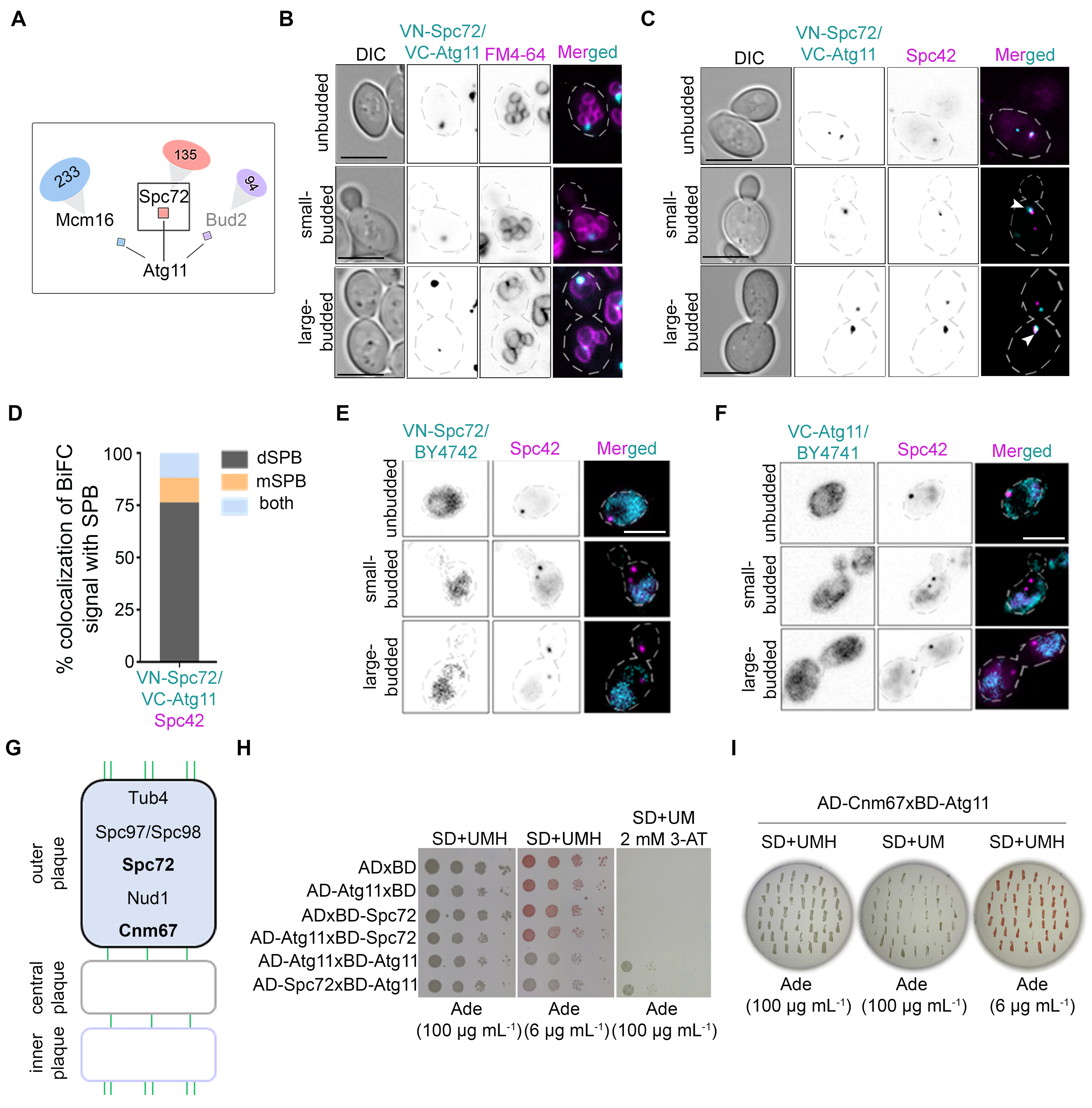

Supplement: S3 Fig — (A) Primary (square boxes) and secondary (oval) interactors of Atg11 as reported in the latest curation of BioGRID [66]. (B) Representative images displaying an in vivo BiFC interaction between Atg11 and Spc72 by the reconstitution of Venus fluorescence (VN-Spc72/VC-Atg11) in diploid cells at the vacuolar periphery, marked by FM4−64 staining. Scale bar, 6 μm. (C) Representative images displaying an in vivo BiFC interaction between Atg11 and Spc72 at the SPBs, (Spc42-mCherry), in the diploid cells. The white arrowheads mark the co-localization of the BiFC signals with the dSPB. Scale bar, 6 μm. (D) A bar diagram displaying the proportion of cells displaying co-localization of BiFC signals with either dSPB, mSPB or both the SPBs (n = 85 large-budded cells). (E) Representative fluorescence images expressing the VN-tagged Spc72 in diploid cells co-expressing Spc42-tagged with mCherry. Scale bar, 6 μm. (F) Representative fluorescence images expressing VC-tagged Atg11 in diploid cells co-expressing Spc42-tagged with mCherry. Scale bar, 6 μm. (G) Schematic showing the spatial position of proteins at the outer plaque of the SPB. (H) Yeast-two hybrid (Y2H) assays in S. cerevisiae strains carrying indicated plasmids were 10-fold serially diluted and spotted on SD - trp - leu or SD - trp - leu - his supplemented with 2 mM 3-amino-1,2,4-triazole (3-AT) and grown at 30 °C. AD- activation domain, BD- DNA binding domain. (I) Yeast-two hybrid (Y2H) assays to study interactions between Atg11 and Cnm67, an outer plaque SPB protein (highlighted in bold in the schematic). The underlying data for panel D can be found in S1 Data. (TIF) [file pbio.3003069.s003.tif]

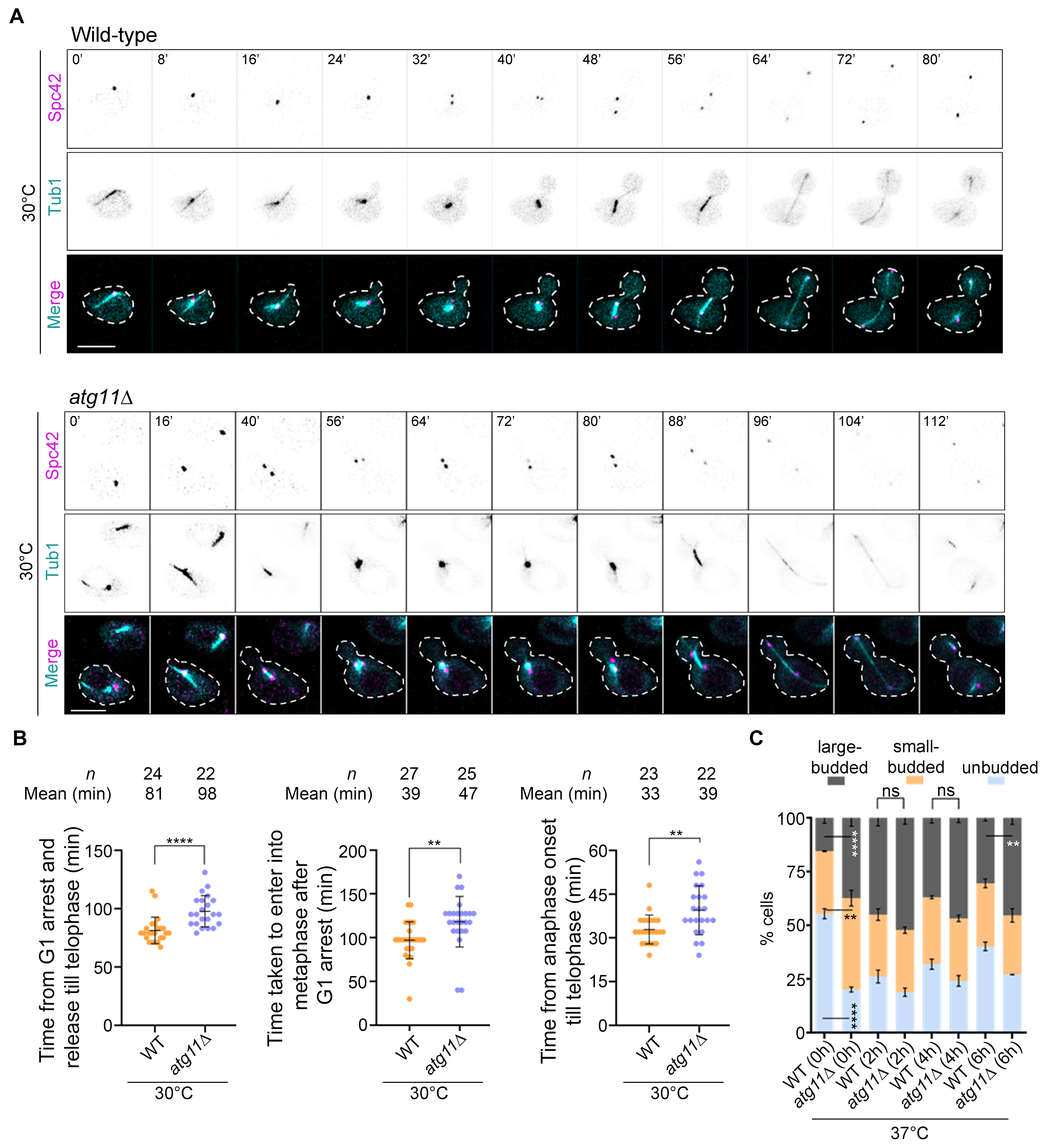

Supplement: S4 Fig — (A) Time-lapse images showing dynamics of the mitotic spindle (GFP-Tub1) and SPBs (Spc42-mCherry) during the cell cycle in wild-type (top) and atg11∆ cells (bottom) after G1 arrest followed by release at 30 °C. Scale bar, 5 μm. (B) Scatter plot displaying time taken for wild-type and atg11∆ cells, after G1 arrest followed by release at 30 °C, for completion of the cell cycle (left), to enter into metaphase (middle, 1.5–2 µm mitotic spindle), and anaphase onset till telophase (right, disassembly of MTs). Error bars show mean ± SEM. Statistical analysis was done using an unpaired t test with Welch’s correction (**p = 0.0045/0.0031, ****p < 0.001). (C) A bar diagram representing the proportion of unbudded, small-budded, and large-budded cells in wild-type (WT) and atg11∆ cells grown in YPD for 0, 2, 4, and 6 h at 37 °C. More than 100 cells were analyzed for each biological replicate and for every time point, N = 3. Error bars show mean ± SEM. Statistical analysis was done by two-way ANOVA using Tukey’s multiple comparisons test (**p = 0.0068/0.0017, ****p < 0.0001). The underlying data for panels B-C can be found in S1 Data. (TIF) [file pbio.3003069.s004.tif]

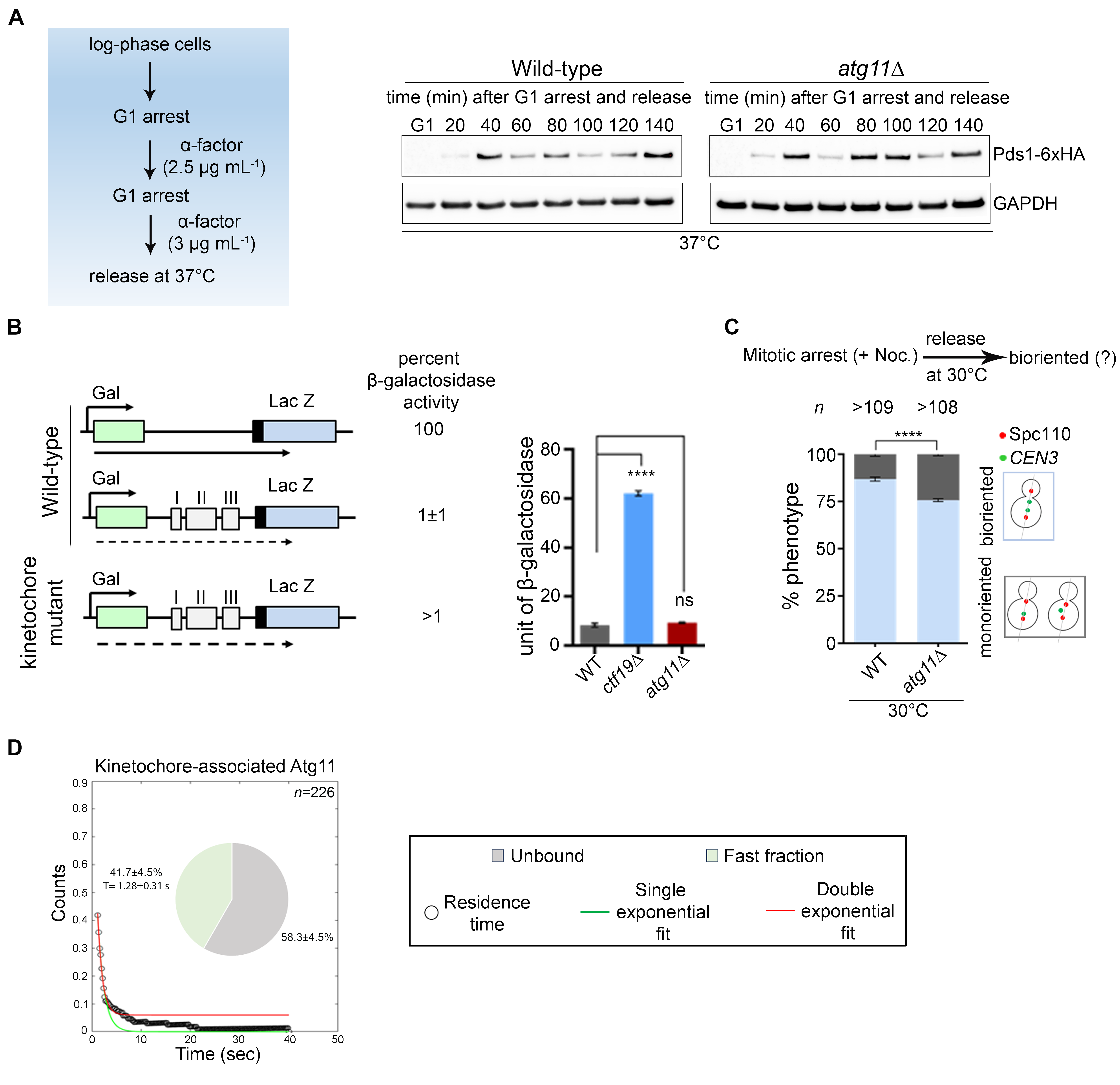

Supplement: S5 Fig — (A) Schematic (left) showing steps involved in synchronizing followed by the release of wild-type and atg11∆ cells to study Pds1 protein dynamics after G1 arrest and release at 37 °C. Western blot analysis (right) shows the expression of Pds1-6xHA in wild-type and atg11∆ cells. Protein levels of GAPDH were used as a loading control. The experiments were repeated twice with similar results. (B) Schematic (left) describing the transcriptional readthrough assay [73]. Briefly, the LacZ (blue) coding sequence is in-frame with the amino-terminal actin ORF (black). CEN DNA (gray), labeled with I, II, and III represent CDE1, CDEII, and CDEIII, respectively. Transcription is under the control of the GAL10 promoter and the solid arrow, below the line diagram, represents maximum activity, while the width of dashed arrows represents β-galactosidase activity. A bar diagram (right) representing β-galactosidase levels of the corresponding strains. The measurement was performed in triplicates. Statistical analysis was done by one-way ANOVA using Dunnett’s multiple comparisons test (p < 0.0001). (C) A bar diagram representing the proportion of cells with bioriented (light blue) or mono-oriented kinetochores (CEN3-GFP) (dark gray) in each strain grown at 30 °C. n represents the minimum number of large-budded cells (budding index of > 0.6) analyzed in three independent biological replicates. Error bars show mean ± SEM. Statistical analysis was done using two-way ANOVA for multiple comparisons (****p < 0.0001). (D) Survival time distribution of Atg11 at kinetochores was quantified from 200 ms time-interval movies. The distribution does not fit well with the double exponential curve, suggesting only a fast fraction. The pie chart represents the percentage of molecules unbound (gray) and bound with short residence time (fast fraction, light green). The average residence time of fast fractions is presented next to their representative fractions. n = number of tracks analyzed. The underlyi [file pbio.3003069.s005.tif]

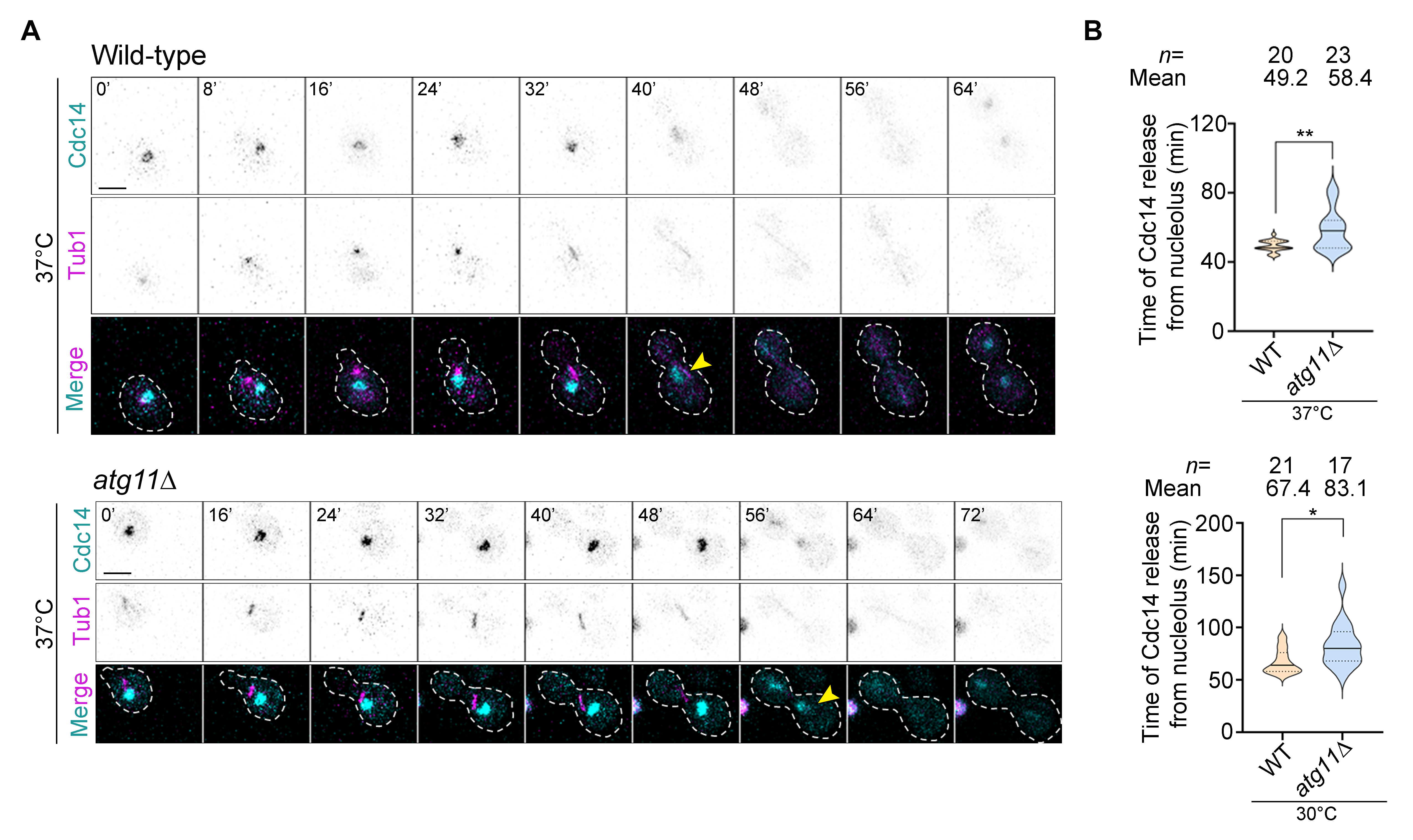

Supplement: S6 Fig — (A) Time-lapse images showing Cdc14 (Cdc14-GFP) and spindle (Tub1-mCherry) dynamics during the cell cycle in wild-type (top) and atg11∆ (bottom) cells after G1 arrest and release at 37 °C. The yellow arrowheads show Cdc14 release from the nucleolus. Scale bar, 5 μm. (B) The violin plot displays the time duration for wild-type and atg11∆ cells to release Cdc14 from the nucleolus, after G1 arrest and release at 37 °C (top) or 30 °C (bottom). Statistical analysis was done using an unpaired t test with Welch’s correction (*p = 0.0108, **p = 0.0014). The underlying data for panel B can be found in S1 Data. (TIF) [file pbio.3003069.s006.tif]

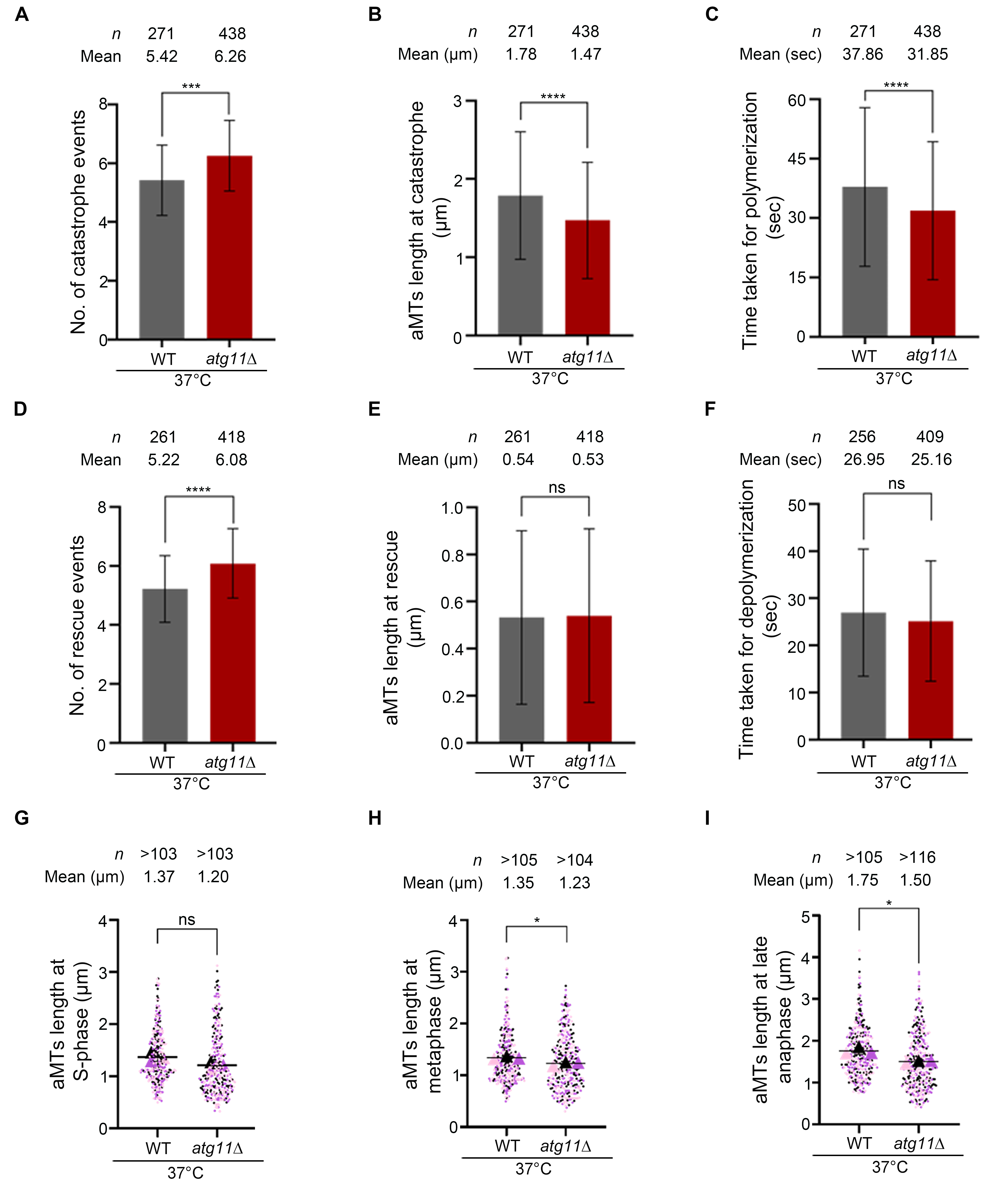

Supplement: S7 Fig — (A) Bar diagram showing the number of catastrophe events in wild-type and atg11∆ cells. (B) Bar diagram displaying aMT length at catastrophe. (C) Bar diagram representing the time taken for polymerization before the catastrophe event. (D) Bar diagram showing the number of rescue events. (E) Bar diagram displaying aMT length at the rescue. (F) Bar diagram representing the time taken for depolymerization before the rescue event. Error bars show mean ± SD. Statistical analysis was done using an unpaired t test with Welch’s correction (***p = 0.0003, ****p < 0.0001). (G) Scatter plot displaying aMT length at S-phase (<1 µm mitotic spindle) in wild-type (WT) and atg11∆ cells grown at 37 °C. (H) Scatter plot representing aMT length at metaphase (1.5–2 µm mitotic spindle) in wild-type (WT) and atg11∆ cells grown at 37 °C. (I) Scatter plot showing aMT length at late anaphase (>7 µm mitotic spindle) in wild-type (WT) and atg11∆ cells grown at 37 °C. n, a minimum number of cells analyzed, N = 3. The statistical significance was done using an unpaired t test with Welch’s correction (*p = 0.0194/0.0191). The underlying data for panels A-I can be found in S1 Data. (TIF) [file pbio.3003069.s007.tif]

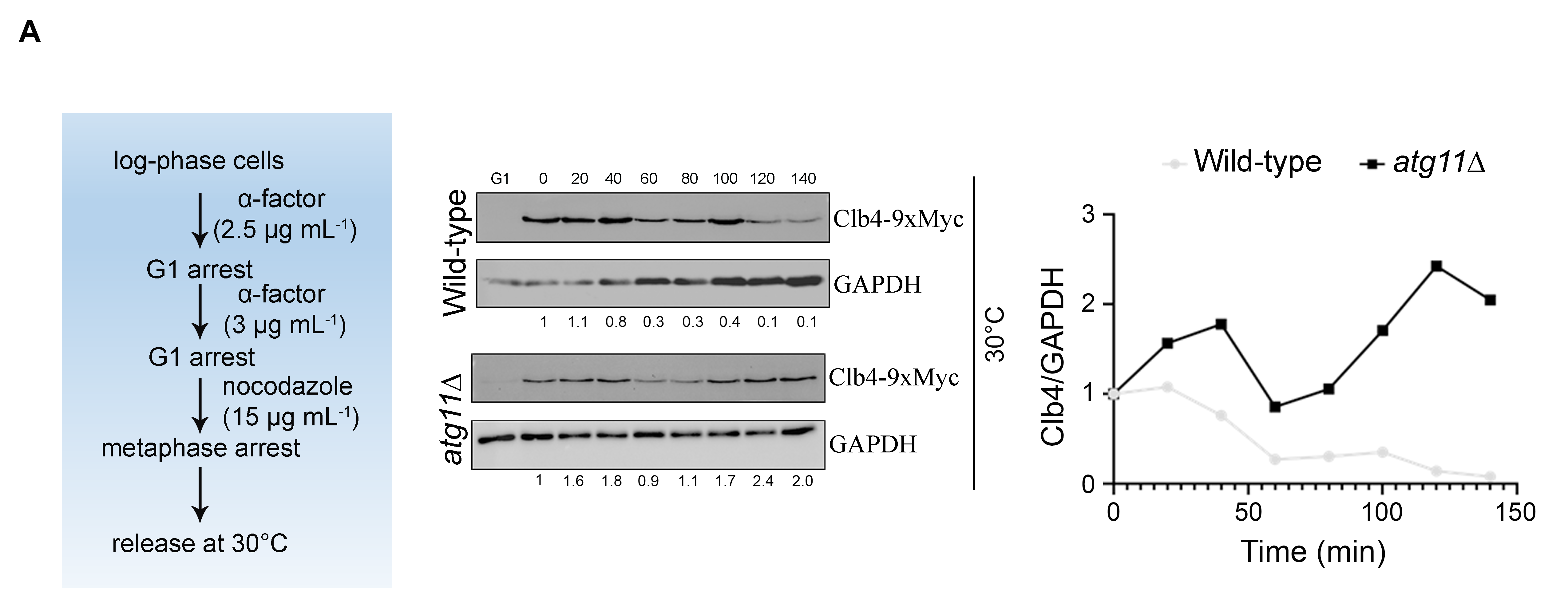

Supplement: S8 Fig — The atg11∆ cells exhibit stabilized levels of Clb4. (A) Schematic (left) showing steps involved in synchronizing followed by the release of wild-type and atg11∆ cells to study Clb4 protein dynamics after G1 and metaphase arrest and release at 30 °C. Cells were collected every 20 min to prepare protein samples. Western blot analysis (right) shows the expression of Clb4-9xMyc in wild-type and atg11∆ cells. Protein levels of GAPDH were used as a loading control. Clb4 normalized values are indicated below each lane and the values are plotted as a line graph. The experiments were repeated twice with similar dynamics. The underlying data for panel A can be found in S1 Data. Uncropped western blots are available in S1 Raw Images. (TIF) [file pbio.3003069.s008.tif]

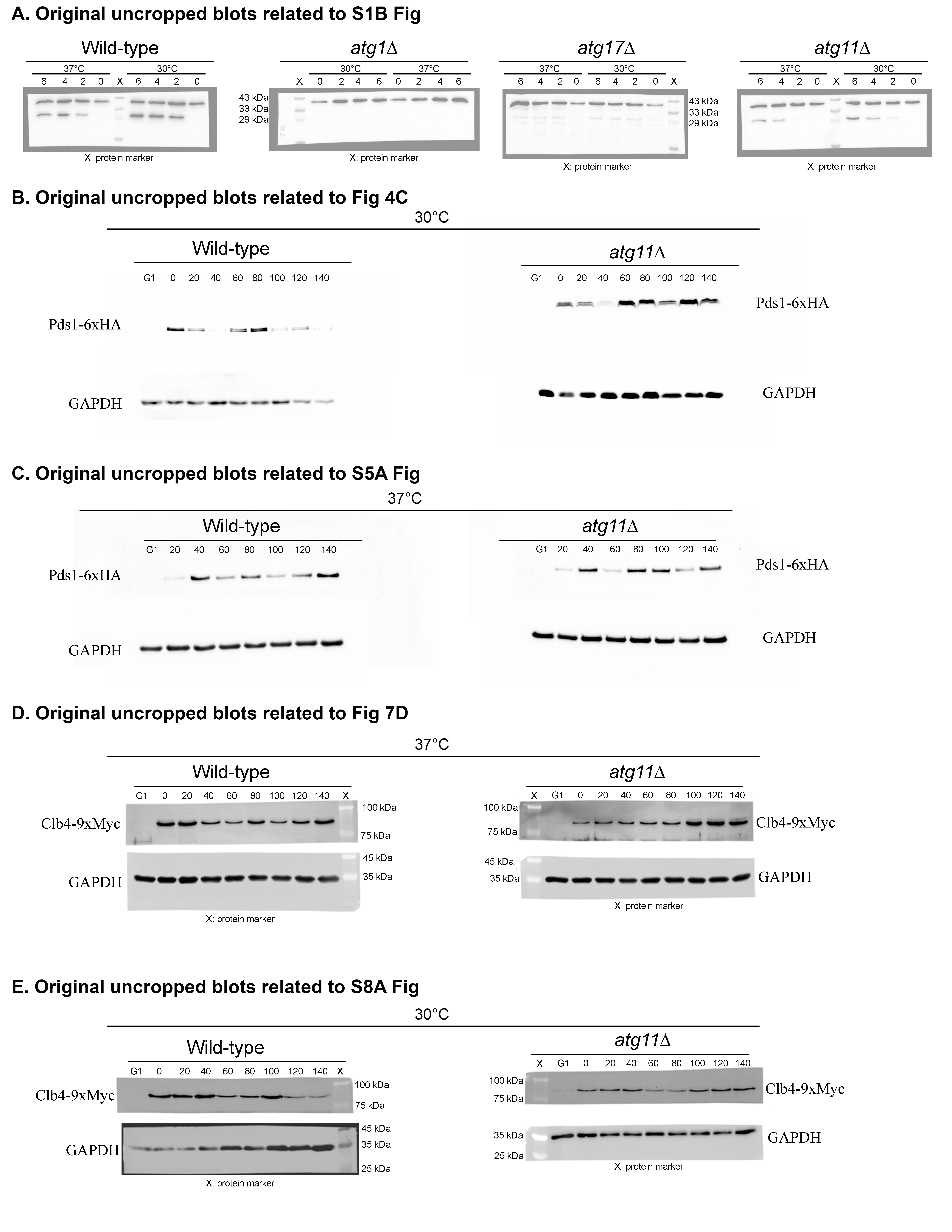

Supplement: S1 Raw Images — Each of the uncropped western blots is shown and overlaid with the protein marker except for western blots shown in S5A Fig. The lane labeled with X represents protein mraker. (TIF) [file pbio.3003069.s013.tif]
