# Supplementary material for: Autophagy-related protein Atg11 is essential for microtubule-mediated chromosome segregation
Source: PLoS Biol. 2025 Apr 2;23(4):e3003069. doi: 10.1371/journal.pbio.3003069 (PMC11984983; doi:10.1371/journal.pbio.3003069)
Supplement: S1 Table — (PDF) [file pbio.3003069.s009.pdf]

Supplementary Table S1: *S. cerevisiae* strains used in this study.

S.No.	Strains	Genotype	Source
1.	BY4741	*MAT***a** *his3Δ1 leu2Δ0 met15Δ0 ura3Δ0*	EUROSCARF
2.	BY4742	*MATα his3Δ1 leu2Δ0 lys2Δ0 ura3Δ0*	EUROSCARF
3.	*atg1Δ*	*MAT***a** *his3Δ1 leu2Δ0 met15Δ0 ura3Δ0 atg1Δ::KanMX4*	EUROSCARF
4.	*atg2Δ*	*MAT***a** *his3Δ1 leu2Δ0 met15Δ0 ura3Δ0 atg2Δ::KanMX4*	EUROSCARF
5.	*atg3Δ*	*MAT***a** *his3Δ1 leu2Δ0 met15Δ0 ura3Δ0 atg3Δ::KanMX4*	EUROSCARF
6.	*atg4Δ*	*MAT***a** *his3Δ1 leu2Δ0 met15Δ0 ura3Δ0 atg4Δ::KanMX4*	EUROSCARF
7.	*atg5Δ*	*MAT***a** *his3Δ1 leu2Δ0 met15Δ0 ura3Δ0 atg5Δ::KanMX4*	EUROSCARF
8.	*atg6Δ*	*MAT***a** *his3Δ1 leu2Δ0 met15Δ0 ura3Δ0 atg6Δ::KanMX4*	EUROSCARF
9.	*atg7Δ*	*MAT***a** *his3Δ1 leu2Δ0 met15Δ0 ura3Δ0 atg7Δ::KanMX4*	EUROSCARF
10.	*atg8Δ*	*MAT***a** *his3Δ1 leu2Δ0 met15Δ0 ura3Δ0 atg8Δ::KanMX4*	EUROSCARF
11.	*atg9Δ*	*MAT***a** *his3Δ1 leu2Δ0 met15Δ0 ura3Δ0 atg9Δ::KanMX4*	EUROSCARF
12.	*atg10Δ*	*MAT***a** *his3Δ1 leu2Δ0 met15Δ0 ura3Δ0 atg10Δ::KanMX4*	EUROSCARF
13.	*atg11Δ*	*MAT***a** *his3Δ1 leu2Δ0 met15Δ0 ura3Δ0 atg11Δ::KanMX4*	EUROSCARF
14.	*atg12Δ*	*MAT***a** *his3Δ1 leu2Δ0 met15Δ0 ura3Δ0 atg12Δ::KanMX4*	EUROSCARF
15.	*atg14Δ*	*MAT***a** *his3Δ1 leu2Δ0 met15Δ0 ura3Δ0 atg14Δ::KanMX4*	EUROSCARF
16.	*atg15Δ*	*MAT***a** *his3Δ1 leu2Δ0 met15Δ0 ura3Δ0 atg15Δ::KanMX4*	EUROSCARF

17.	*atg16Δ*	*MAT***a** *his3Δ1 leu2Δ0 met15Δ0 ura3Δ0 atg16Δ::KanMX4*	EUROSCARF
18.	*atg17Δ*	*MAT***a** *his3Δ1 leu2Δ0 met15Δ0 ura3Δ0 atg17Δ::KanMX4*	EUROSCARF
19.	*atg18Δ*	*MAT***a** *his3Δ1 leu2Δ0 met15Δ0 ura3Δ0 atg18Δ::KanMX4*	EUROSCARF
20.	*atg19Δ*	*MAT***a** *his3Δ1 leu2Δ0 met15Δ0 ura3Δ0 atg19Δ::KanMX4*	EUROSCARF
21.	*atg20Δ*	*MAT***a** *his3Δ1 leu2Δ0 met15Δ0 ura3Δ0 atg20Δ::KanMX4*	EUROSCARF
22.	*atg21Δ*	*MAT***a** *his3Δ1 leu2Δ0 met15Δ0 ura3Δ0 atg21Δ::KanMX4*	EUROSCARF
23.	*atg22Δ*	*MAT***a** *his3Δ1 leu2Δ0 met15Δ0 ura3Δ0 atg22Δ::KanMX4*	EUROSCARF
24.	*atg23Δ*	*MAT***a** *his3Δ1 leu2Δ0 met15Δ0 ura3Δ0 atg23Δ::KanMX4*	EUROSCARF
25.	*atg24Δ*	*MAT***a** *his3Δ1 leu2Δ0 met15Δ0 ura3Δ0 atg24Δ::KanMX4*	EUROSCARF
26.	*atg26Δ*	*MAT***a** *his3Δ1 leu2Δ0 met15Δ0 ura3Δ0 atg26Δ::KanMX4*	EUROSCARF
27.	*atg27Δ*	*MAT***a** *his3Δ1 leu2Δ0 met15Δ0 ura3Δ0 atg27Δ::KanMX4*	EUROSCARF
28.	*atg29Δ*	*MAT***a** *his3Δ1 leu2Δ0 met15Δ0 ura3Δ0 atg29Δ::KanMX4*	EUROSCARF

29.	*atg31Δ*	*MAT***a** *his3Δ1 leu2Δ0 met15Δ0 ura3Δ0 atg31Δ::KanMX4*	EUROSCARF
30.	*atg32Δ*	*MAT***a** *his3Δ1 leu2Δ0 met15Δ0 ura3Δ0 atg32Δ::KanMX4*	EUROSCARF
31.	*atg33Δ*	*MAT***a** *his3Δ1 leu2Δ0 met15Δ0 ura3Δ0 atg33Δ::KanMX4*	EUROSCARF
32.	*atg34Δ*	*MAT***a** *his3Δ1 leu2Δ0 met15Δ0 ura3Δ0 atg34Δ::KanMX4*	EUROSCARF
33.	*atg36Δ*	*MAT***a** *his3Δ1 leu2Δ0 met15Δ0 ura3Δ0 atg36Δ::KanMX4*	EUROSCARF
34.	*atg38Δ*	*MAT***a** *his3Δ1 leu2Δ0 met15Δ0 ura3Δ0 atg38Δ::KanMX4*	EUROSCARF
35.	*atg39Δ*	*MAT***a** *his3Δ1 leu2Δ0 met15Δ0 ura3Δ0 atg39Δ::KanMX4*	EUROSCARF
36.	*atg40Δ*	*MAT***a** *his3Δ1 leu2Δ0 met15Δ0 ura3Δ0 atg40Δ::KanMX4*	EUROSCARF
37.	*atg41Δ*	*MAT***a** *his3Δ1 leu2Δ0 met15Δ0 ura3Δ0 atg41Δ::KanMX4*	EUROSCARF
38.	*ctf19Δ*	*MAT***a** *his3Δ1 leu2Δ0 met15Δ0 ura3Δ0 ctf19Δ::KanMX4*	EUROSCARF
39.	*mcm16Δ*	*MAT***a** *his3Δ1 leu2Δ0 met15Δ0 ura3Δ0 mcm16Δ::KanMX4*	EUROSCARF
40.	JV01	*MAT***a** *his3Δ1 leu2Δ0 met15Δ0 ura3Δ0* pRS313 (*HIS3*)	This study
41.	JV02	*MAT***a** *his3Δ1 leu2Δ0 met15Δ0 ura3Δ0 ctf19Δ::KanMX4* pRS313 (*HIS3*)	This study

42.	JV03	*MAT***a** *his3Δ1 leu2Δ0 met15Δ0 ura3Δ0 atg1Δ::KanMX4* pRS313 (*HIS3*)	This study
43.	JV04	*MAT***a** *his3Δ1 leu2Δ0 met15Δ0 ura3Δ0 atg6Δ::KanMX4* pRS313 (*HIS3*)	This study
44.	JV05	*MAT***a** *his3Δ1 leu2Δ0 met15Δ0 ura3Δ0 atg11Δ::KanMX4* pRS313 (*HIS3*)	This study
45.	JV06	*MAT***a** *his3Δ1 leu2Δ0 met15Δ0 ura3Δ0 atg15Δ::KanMX4* pRS313 (*HIS3*)	This study
46.	JV07	*MAT***a** *his3Δ1 leu2Δ0 met15Δ0 ura3Δ0 atg17Δ::KanMX4* pRS313 (*HIS3*)	This study
47.	JV08	*MAT***a** *his3Δ1 leu2Δ0 met15Δ0 ura3Δ0::URA3 GAL1-10-actin-CEN4-lacZ*	This study
48.	JV09	*MAT***a** *his3Δ1 leu2Δ0 met15Δ0 ura3Δ0::URA3 GAL1-10-actin-CEN4-lacZ ctf19Δ::KanMX4*	This study
49.	JV10	*MAT***a** *his3Δ1 leu2Δ0 met15Δ0 ura3Δ0::URA3 GAL1-10-actin-CEN4-lacZ atg11Δ::KanMX4*	This study
50.	YMH58a	*MAT***a** *15ade2-1 ura3-1 112 his 3-11 trp1-1 leu2-3, CFIII (CEN3.L.YMH58) URA3 SUP11*	A gift from Sunanda Bhattacharyya
51.	MHR01	*MAT***a** *his3Δ1 leu2Δ0 met15Δ0 ura3Δ0::URA3* GFP-*TUB1*	This study
52.	MHR02	*MAT***a** *his3Δ1 leu2Δ0 met15Δ0 ura3Δ0::URA3* GFP-*TUB1 atg11Δ::KanMX4*	This study
53.	MHR08	*MATα his3Δ1 leu2Δ0 lys2Δ0 ura3Δ0 atg11Δ::HPH*	This study

54.	MHR14	*MATa his3Δ1 leu2Δ0 met15Δ0 ura3Δ0 dyn1Δ::KanMX4 atg11Δ::HPH GFP-TUB1::URA3*	This study
55.	MHR19	*MAT***a** *15ade2-1 ura3-1 112 his 3-11 trp1-1 leu2-3 CFIII (CEN3.L.YMH58) URA3 SUP11 atg11Δ::HPH*	This study
56.	MHR35	*MAT***a** *his3Δ1 leu2Δ0 met15Δ0 ura3Δ0::URA3* GFP-*TUB1* Spc42-mCherry*::KanMX4*	This study
57.	MHR52	*MAT***a** *leu2Δ0 met15Δ0 ura3Δ0 atg11Δ::KanMX4 his3Δ1::HIS3 ATG11*	This study
58.	PJ69-4A	*MAT***a** *trp1-901 leu2-3, 112 ura3-52 his3-200 gal4Δ gal80Δ LYS2::GAL1-HIS3 GAL1-ADE2 met2::GAL7-lacZ*	(1)
59.	MHR54	*MAT***a** *trp1-901 leu2-3, 112 ura3-52 his3-200 gal4Δ gal80Δ LYS2::GAL1-HIS3 GAL1-ADE2 met2::GAL7-lacZ* pGADC1 (*LEU2*) pGBDC1 (*TRP1*)	This study
60.	MHR57	*MAT***a** *trp1-901 leu2-3, 112 ura3-52 his3-200 gal4Δ gal80Δ LYS2::GAL1-HIS3 GAL1-ADE2 met2::GAL7-lacZ ATG11::LEU2* (pGADC1) pGBDC1 (*TRP1*)	This study
61.	MHR60	*MAT***a** *trp1-901 leu2-3, 112 ura3-52 his3-200 gal4Δ gal80Δ LYS2::GAL1-HIS3 GAL1-ADE2 met2::GAL7-lacZ* pGADC1 (*LEU2*) *SPC72::TRP1* (pGBDC1)	This study
62.	MHR63	*MAT***a** *trp1-901 leu2-3, 112 ura3-52 his3-200 gal4Δ gal80Δ LYS2::GAL1-HIS3 GAL1-ADE2 met2::GAL7-lacZ ATG11::LEU2* (pGADC1) *SPC72::TRP1* (pGBDC1)	This study

63.	MHR66	*MAT***a** *trp1-901 leu2-3, 112 ura3-52 his3-200 gal4Δ gal80Δ LYS2::GAL1-HIS3 GAL1-ADE2 met2::GAL7-lacZ ATG11::LEU2* (pGADC1) *ATG11::TRP1* (pGBDC1)	This study
64.	MHR69	*MAT***a** *trp1-901 leu2-3, 112 ura3-52 his3-200 gal4Δ gal80Δ LYS2::GAL1-HIS3 GAL1-ADE2 met2::GAL7-lacZ SPC72::LEU2* (pGADC1) *ATG11::TRP1* (pGBDC1)	This study
65.	MHR72	*MAT***a** *trp1-901 leu2-3, 112 ura3-52 his3-200 gal4Δ gal80Δ LYS2::GAL1-HIS3 GAL1-ADE2 met2::GAL7-CNM67::LEU2* (pGADC1) *ATG11::TRP1* (pGBDC1)	This study
66.	MHR76	*MAT***a** *his3Δ1 leu2Δ0 met15Δ0 ura3Δ0::URA3* GFP-*TUB1 kar9Δ::KanMX4*	This study
67.	MHR80	*MAT***a** *his3Δ1 leu2Δ0 met15Δ0 ura3Δ0 atg11Δ::KanMX4*	This study
68.	yTB283	*MAT***a** *his3Δ1 leu2Δ0::LEU2* sfGFP-*ATG11 met15Δ0 ura3Δ0*	(2)
69.	MHR90	*MAT***a** *his3Δ1 leu2Δ0::LEU2* sfGFP-*ATG11 met15Δ0 ura3Δ0* Spc42-mCherry*::KanMX4*	This study
70.	*dyn1Δ*	*MAT***a** *his3Δ1 leu2Δ0 met15Δ0 ura3Δ0 dyn1Δ::KanMX4*	EUROSCARF
71.	*kip2Δ*	*MAT***a** *his3Δ1 leu2Δ0 met15Δ0 ura3Δ0 kip2Δ::KanMX4*	EUROSCARF
72.	MHR174	*MAT***a** *15ade2-1 ura3-1 112 his 3-11 trp1-1 leu2-3 CFIII (CEN3.L.YMH58) URA3 SUP11 ctf19Δ::HPH*	This study
73.	MHR189	*MAT***a** *his3Δ1 leu2Δ0 met15Δ0 ura3Δ0::URA3* GFP-*TUB1 atg1Δ::HPH* Spc42-mCherry*::KanMX4*	This study

74.	MHR200	*MAT***a** *his3Δ1 leu2Δ0 met15Δ0 ura3Δ0::URA3* GFP-*TUB1 atg11Δ::HPH kar9Δ::KanMX4*	This study
75.	MHR202	*MAT***a** *his3Δ1 leu2Δ0 met15Δ0 ura3Δ0::URA3* GFP-*TUB1 dyn1Δ::KanMX4 H1*-mCherry::*LEU2*	This study
76.	MHR205	*MAT***a** *his3Δ1 leu2Δ0 met15Δ0 ura3Δ0::URA3* GFP-*TUB1 atg11Δ::HPH dyn1Δ::KanMX4 H1*-mCherry::*LEU2*	This study
77.	MHR209	*MAT***a** *his3Δ1 leu2Δ0 met15Δ0 ura3Δ0::URA3* GFP-*TUB1 kip2Δ::HPH atg11Δ::KanMX4 H1*-mCherry::*LEU2*	This study
78.	MHR210	*MAT***a** *his3Δ1 leu2Δ0 met15Δ0 ura3Δ0::URA3* GFP-*TUB1 kip2Δ::HPH dyn1Δ::KanMX4 H1*-mCherry::*LEU2*	This study
79.	MHR211	*MAT***a** *his3Δ1 leu2Δ0 met15Δ0 ura3Δ0::URA3* GFP-*TUB1 kip2Δ::HPH H1*-mCherry::*LEU2*	This study
80.	*kar9Δ*	*MAT***a** *his3Δ1 leu2Δ0 met15Δ0 ura3Δ0 kar9Δ::KanMX4*	EUROSCARF
81.	MHR216	*MAT*a *his3Δ1 leu2Δ0 met15Δ0 ura3Δ0::URA3* GFP-*TUB1 kar9Δ::HIS3* Spc42-mCherry*::KanMX4*	This study
82.	F2351	W303**a** *ade2-1oc ade3Δ-100 can1-100 cyh2R his3-11,15 leu2-3,112 trp1-1 ura3-1 LYS2 bar1-1 SPC72-Cherry::KanMx6 KAR9-GFP::HIS3Mx6*	(3)
83.	MHR221	W303**a** *ade2-1oc ade3Δ-100 can1-100 cyh2R his3-11,15 leu2-3,112 trp1-1 ura3-1 LYS2 bar1-1 SPC72-Cherry::KanMx6 KAR9-GFP::HIS3Mx6 atg11Δ::HPH*	This study

84.	SBY12503	*MAT***a** *pCUP1-GFP12-LacI12:HIS3 CEN3::33LacO:KanMX SPC110-mCherry:hphMX HSK3-3V5-IAA7:KanMX bar1-1 ade3Δ*	(4)
85.	MHR227	*MAT***a** *pCUP1-GFP12-LacI12:HIS3 CEN3::33LacO:KanMX SPC110-mCherry:hphMX HSK3-3V5-IAA7:KanMX bar1-1 ade3Δ atg11Δ::LEU2*	This study
86.	MHR251	*MAT***a***/α his3Δ1/his3Δ1 leu2Δ0/leu2Δ0 met15Δ0/MET15 lys2Δ0/LYS2 ura3Δ0/ura3Δ0* VN-*SPC72::KanMX4* x VC-*ATG11::HIS3*	This study
87.	MHR279	*MAT***a***/α his3Δ1/his3Δ1 leu2Δ0/leu2Δ0 met15Δ0/MET15 lys2Δ0/LYS2 ura3Δ0/ura3Δ0* VN-*SPC72::KanMX4* x VC-*ATG11::HIS3* Spc42-mCherry*::KanMX4*	This study
88.	MHR282	*MAT***a***/α his3Δ1/ his3Δ1 leu2Δ0/ leu2Δ0 met15Δ0/MET15 lys2Δ0/LYS2 ura3Δ0/ ura3Δ0* VC-*ATG11::HIS3* Spc42-mCherry*::KanMX4*	This study
89.	MHR284	*MAT***a***/α his3Δ1/ his3Δ1 leu2Δ0/ leu2Δ0 met15Δ0/MET15 lys2Δ0/LYS2 ura3Δ0/ ura3Δ0* VN-*SPC72::KanMX4* Spc42-mCherry*::KanMX4*	This study
90.	MHR285	*MAT***a** *his3Δ1 leu2Δ0 met15Δ0 ura3Δ0::URA3* GFP-*TUB1 atg11Δ::HPH* Spc42-mCherry*::KanMX4*	This study
91.	MHR348	*MATα his3Δ1 leu2Δ0 lys2Δ0 ura3Δ0::URA3 ATG11-*Halo *pdr5Δ0::LoxP*	This study
92.	MHR350	*MAT***a** *his3Δ1 leu2Δ0::LEU2* sfGFP-*ATG11 met15Δ0 ura3Δ0 pdr5Δ::HPH* Spc42-mCherry*::KanMX4*	This study

93.	MHR351	*MAT***a**/*α his3Δ1/his3Δ1 leu2Δ0/LEU2* sfGFP-*ATG11 met15Δ0/MET15 lys2Δ0/LYS2 ura3Δ0/URA3 ATG11-*Halo Spc42-mCherry*::KanMX4 pdr5Δ::HPH/pdr5Δ0::LoxP*	
94.	MHR437	*MATα his3Δ1 leu2Δ0 lys2Δ0 ura3Δ0 mad2Δ::HPH*	This study
95.	MHR442	*MATα his3Δ1 leu2Δ0 ura3Δ0 MET15 LYS2 atg11Δ::KanMX4 mad2Δ::HPH*	This study
96.	MHR493	*MAT***a** *ura3-52 leu2Δ1 trp1Δ63 his3Δ200* Cdc14-GFP::HPH Tub1-mCherry::*URA3 atg11Δ::LEU2*	This study
97.	ScRA403	*MATa his3Δ1 leu2Δ0 met15Δ0 ura3Δ0* Pds1-6xHA::*HPH* Clb2-9xMyc*::HIS3*	This study
98.	ScRA405	*MAT***a** *his3Δ1 leu2Δ0 met15Δ0 ura3Δ0 lys2Δ0 atg11Δ::HPH* Pds1-6xHA*::HPH* Clb2-9xMyc*::HIS3*	This study
99.	ScRA413	*MAT***a** *trp1-901 leu2-3, 112 ura3-52 his3-200 gal4Δ gal80Δ LYS2::GAL1-HIS3 GAL1-ADE2 met2::GAL7-lacZ SPC72::LEU2* (pGADC1) *ATG11*L867G*::TRP1* (pGBDC1)	This study
100.	ScRA414	*MAT***a** *trp1-901 leu2-3, 112 ura3-52 his3-200 gal4Δ gal80Δ LYS2::GAL1-HIS3 GAL1-ADE2 met2::GAL7-lacZ SPC72::LEU2* (pGADC1) *ATG11*D1001A*::TRP1* (pGBDC1)	This study
101.	ScRA415	*MAT***a** *trp1-901 leu2-3, 112 ura3-52 his3-200 gal4Δ gal80Δ LYS2::GAL1-HIS3 GAL1-ADE2 met2::GAL7-lacZ*	This study

		SPC72::LEU2 (pGADC1) *ATG11*L1005G*::TRP1* (pGBDC1)	
102.	ScRA417	*MAT***a** *his3Δ1 leu2Δ0 met15Δ0 ura3Δ0* VN-*SPC72::KanMX4*	This study
103.	ScRA431	*MATα his3Δ1 leu2Δ0 lys2Δ0 ura3Δ0* VC-*ATG11::HIS3*	This study
104.	ESM356-1	*MAT***a** *leu2Δ1 trp1Δ63 his3Δ200 ura3-52*	(5)
105.	YSP648	*MAT***a** *leu2Δ1 trp1Δ63 his3Δ200 ura3-52* Cdc14-GFP*::hphNT1*	Dr. Saravanan Palani, IISc
106.	YSP650	*MAT***a** *leu2Δ1 trp1Δ63 his3Δ200 ura3-52::URA3*-mCherry-Tub1 Cdc14-GFP::*hphNT1*	Dr. Saravanan Palani, IISc
107.	SBY322	*MAT***a** *ura3-1 leu2-3,112 his3-11:pCUP1-GFP12-lacI12:HIS3 trp1-1:lacO:TRP1 lys2Δ ade2-1 bar1Δ can1-100 ipl1-321*	(6)
108.	MHR504	*MAT***a** *ura3-1 leu2-3,112 his3-11:pCUP1-GFP12-lacI12:HIS3 trp1-1:lacO:TRP1 lys2Δ ade2-1 bar1Δ can1-100 ipl1-321 atg11Δ::HPH*	This study
109.	MHR411	*MAT***a** *his3Δ1 leu2Δ0::LEU2* sfGFP-*ATG11 met15Δ0 ura3Δ0 pdr5Δ::HPH*	This study
110.	MHR502	*MAT***a** *his3Δ1 leu2Δ0::LEU2* sfGFP-*ATG11 met15Δ0 ura3Δ0 pdr5Δ::HPH* Mtw1-mCherry*::KanMX4*	This study
111.	MHR505	*MAT***a**/*α his3Δ1/his3Δ1 leu2Δ0/LEU2* sfGFP-*ATG11 met15Δ0/MET15 lys2Δ0/LYS2 ura3Δ0/URA3 ATG11*-Halo Mtw1-mCherry*::KanMX4 pdr5Δ::HPH/pdr5Δ0::LoxP*	This study

112.	MHR509	*MAT***a** *his3Δ1 leu2Δ0 met15Δ0 ura3Δ0* Clb4-9xMyc*::HIS3*	This study
113.	MHR511	*MAT***a** *his3Δ1 leu2Δ0 met15Δ0 ura3Δ0 atg11Δ::KanMX4* Clb4-9xMyc*::HIS3*	This study

References

1. James P, Halladay J, Craig EA. Genomic libraries and a host strain designed for highly efficient two-hybrid selection in yeast. Genetics. 1996;144(4):1425-36.

2. Pfaffenwimmer T, Reiter W, Brach T, Nogellova V, Papinski D, Schuschnig M, et al. Hrr25 kinase promotes selective autophagy by phosphorylating the cargo receptor Atg19. EMBO Rep. 2014;15(8):862-70.

3. Manzano-Lopez J, Matellan L, Alvarez-Llamas A, Blanco-Mira JC, Monje-Casas F. Asymmetric inheritance of spindle microtubule-organizing centres preserves replicative lifespan. Nat Cell Biol. 2019;21(8):952-65.

4. Umbreit NT, Miller MP, Tien JF, Ortola JC, Gui L, Lee KK, et al. Kinetochores require oligomerization of Dam1 complex to maintain microtubule attachments against tension and promote biorientation. Nat Commun. 2014;5:4951.

5. Pereira G, Tanaka TU, Nasmyth K, Schiebel E. Modes of spindle pole body inheritance and segregation of the Bfa1p-Bub2p checkpoint protein complex. EMBO J. 2001;20(22):6359-70.

6. Buvelot S, Tatsutani SY, Vermaak D, Biggins S. The budding yeast Ipl1/Aurora protein kinase regulates mitotic spindle disassembly. The Journal of cell biology. 2003;160(3):329-39.
