# Supplementary material for: Autophagy-related protein Atg11 is essential for microtubule-mediated chromosome segregation
Source: PLoS Biol. 2025 Apr 2;23(4):e3003069. doi: 10.1371/journal.pbio.3003069 (PMC11984983; doi:10.1371/journal.pbio.3003069)
Supplement: S2 Table — (PDF) [file pbio.3003069.s010.pdf]

Supplementary Table S2: Oligonucleotides used in this study.

Primer Name	Sequence (5' to 3')	Application
HR34	GTTCGGAAAGTACTTCTTTTATTTTCTTTTATA CATCATGCGGATCCCCGGGTTAATTAA	For *ATG11* deletion cassette amplification having *HPH* marker
HR35	AAATCTTGTCATTTGTCACAAACGTTTAGCACT GTTCAAACCGACAGCAGTATAGCGACCA	
HR36	GAGTACGGCTCTGATAGATC	5' and 3' Junction for confirming *ATG11* deletion
HR37	CAAACTCCTCTTCGGTTGTAG	
HR39	CACAGTTTGCCAGTGATACAC	*HPH*_Reverse
HR40	CTCCAACAATGTCCTGACGGAC	*HPH*_Forward
HR52	TTGAAAACCACTTCAAAGGGGCCCAATAGCAC ATTTAATCGGATCCCC	For *MAD2* deletion cassette amplification having *HPH* marker
HR53	TTGACTTGAATTCTATTAATATTTATAGCTGAC CTGCGCACGACAGCAGTATAG	
HR58	GTGTGATCTTGTTGATACTAGGTCGGCAAAGA ACGCAAATCGGATCCCCGGGTTAATTAAG	For *CTF19* deletion cassette amplification having *HPH* marker
HR59	GTTTAAGCAAGCCGTCCAGTTGGCAATGGCAA ATGGAACACGACAGCAGTATAGCGACCAG	
HR60	CGTTCTACTATGTGGCCGG	5' and 3' Junction for confirming *CTF19* deletion
HR61	GTTCATATGATAAACGACGATG	
HR66	CGCCCCGAAGAATGATTGATG	5' and 3' Junction for confirming *MAD2* deletion
HR67	GTTTACAGAGGAAGTACGTAG	

HR86	CCGGCATTATTAAACTAAACAAGAAGAAGGTCAAACTCTCCACGATGGTGAGCAAGGGCGAGG	mCherry_Forward
HR87	GGACAACGATATTCTTAGACATCTTGTACAGCTCGTCCATG	mCherry_Reverse
HR88	CATGGACGAGCTGTACAAGATGTCTAAGAATATCGTTGTCC	*LEU*_Forward
HR89	GTATTGCTATCACCATTGACATTCTCGTTTGGATATTCACTTTAGCCAAGATTTCCTTGACAGC	*LEU*_Reverse
HR90	CCGGCATTATTAAACTAAACAAG	5' and 3' Junction for the full-length amplification of mCherry-*LEU2*
HR91	GTATTGCTATCACCATTGACATTC	
HR116	CACACTATGCAACCAATGGG	5' and 3' Junction for confirming *ATG11* deletion using *KanMX4* as a marker
HR117	AGAGGGGGAACAGATAAGG	
HR131	GAGAGTGTGGATCCATGGCAGACGCTGATGAATATAG	For cloning *ATG11* in pGADC1/ pGBDC1 for yeast-two hybrid assay
HR132	GAGAGTGTTGTCGACTCAAACTCCCTGGTATGAAACC	
HR133	GAGAGTGTGGATCCATGGTACGTCGATGGATTCC	For cloning *SPC72* in pGADC1/ pGBDC1 for yeast-two hybrid assay
HR134	GAGAGTGTTGTCGACTTAGGGATTGTTGATTGATAGG	
HR135	CATAAAAAAATATAGAGTGTACTAGTCAAACTCCCTGGTATGAAACCAC	For *atg11*Δ null mutant complementation using full-

HR136	TGTGGTTTCATACCAGGGAGTTTGACTAGTACACTCTATATTTTTTTATG	length *ATG11* with *HIS3* marker
HR137	AATTAAAATCTTGTCATTTGTGACAAACGTTTAGCACTGTCTACATAAGAACACCTTTGGTGG	
HR138	TTCAAATCTCTTTTACAACACCAGACGAGAAATTAAGAAACGGATCCCCGGGTTAATTAAG	For *ATG1* deletion cassette amplification having *HPH* marker
HR139	GGTCATTTGTACTTAATAAGAAAACCATATTATGCATCACCGACAGCAGTATAGCGACCAG	
HR140	CATAAGGCAAAGGAGATAGGAG	5’ and 3’ Junction for confirming *ATG1* deletion
HR141	GCATTTCGAGAGTAGCATAAC	
HR142	GCTGAAGCGTGTCGAAGAAG	For *SPC42* tagging with mCherry using *KanMX4* marker
HR143	CATTGGAACCGCAGATTGCTAG	
HR147	GGTAGCATAAACAAATAACATTG	5’ and 3’ Junction for confirming *KAR9* deletion
HR148	GCGTCGGGTAAAGGTGTC	
HR196	GAGAGTGTGGATCCATGACTGATTTCGATTTAATGAATTT	For cloning *CNM67* in pGADC1 for yeast-two hybrid assay
HR197	GAGAGTGTTGTCGACTTACTTCACCATCTTCTCCAGG	
HR200	CTTTGTCTGTAACAGCCTTAAAGATTTCAGTAGCACTGCCCTAGTACACTCTATATTTTTTTATGC	For *KAR9* deletion cassette amplification having *HIS3* marker
HR201	GATATATAAAAATGTATAAGTATACAGTTTTAGGTTAGTACTACATAAGAACACCTTTGGTGG	

HR235	GTTGTTCGGAAAGTACTTCTTTTATTTTCTTTT ATACATCATGTCTAAGAATATCGTTGTCCT	For *ATG11* deletion cassette amplification having *LEU2* marker
HR236	CATAATTAAAATCTTGTCATTTGTGACAAACG TTTAGCACTTAAGCCAAGATTTCCTTGACAG	
HR296	ATGGAACTATATAATAGCGATGCCCTTATTTG AGTACCATGAATTCGAGCTCGTTTAAAC	Used for N-terminal tagging of Atg11, having VN (HR296+HR297) and VC (HR296+HR298) fragments, for bimolecular fluorescence complementation (BiFC) assay
HR297	GCTGGGTAGGTGCAGTGCTATATTCATCAGCG TCTGCCATAGTACCACCAGAACCCTCGATGTT GTGGCGGATC	
HR298	GCTGGGTAGGTGCAGTGCTATATTCATCAGCG TCTGCCATAGTACCACCAGAACCCTTGTACAG CTCGTCCATG	
HR299	ATCACTTTTAGTTTACTTTTGCTTCCCTCTGTGT ATATTCGAATTCGAGCTCGTTTAAAC	Used for N-terminal tagging of Spc72, having VN (HR299+HR300) and VC (HR299+HR301) fragments, for bimolecular fluorescence complementation (BiFC) assay
HR300	TGCGAAGATGCCTGCCACTAGGAATCCATCGA CGTACCATAGTACCACCAGAACCCTCGATGTT GTGGCGGATC	
HR301	TGCGAAGATGCCTGCCACTAGGAATCCATCGA CGTACCATAGTACCACCAGAACCCTTGTACAG CTCGTCCATG	
HR329	GCTCAGTTTGGTTTCAGGTCACTGCGGTTGTG GTTTCATACCAGGGAGTTCAAGCGGCCGCCGC TGCTGC	Used for C-terminal tagging of Atg11 with Halo tag

HR330	GATACATAATTAAAATCTTGTCATTTGTGACAAACGTTTAGCACTGTTCAGTTACTTGGTTCTGGCGAGG	
HR333	CTTTTAAGTTTTCGTATCCGCTCGTTCGAAAGACTTTAGACCGGATCCCCGGGTTAATTAAG	For *PDR5* deletion cassette amplification having *HPH* marker
HR334	CATCTTGGTAAGTTTCTTTTCTTAACCAAATTCAAAATTCCGACAGCAGTATAGCGACCAG	
HR335	TTGGCAACTAGGAACTTTCG	5' and 3' Junction for confirming *PDR5* deletion
HR336	GCACCTATATGTAGTGATTATG	
RA140:Pds1_S3	AGCGAAGAAGGCCTCGATCCTGAAGAACTAGAGGACTTAGTTACTCGTACGCTGCAGGTCGAC	Used for Pds1 tagging
RA141:Pds1_S2	ATACGTGTATATATGTTGTGTGTATGTGAATGAGCAGTGGATCTAATCGATGAATTCGAGCTCG	
RA146: HygR_RP	TTACCCGCAGGACATATCCAC	
RA177	TATTGAAGAGCCTCAATTGGATTTACTTGATGATGTGTTAGGTGCTATGGTGAGCAAGGGCGAG	Used for tagging Mtw1 with mCherry at C-terminus
RA178	CATACATCATATCATAGCACATACTTTTTCCCACTTTATAGCGGCGTTAGTATCGAATCG	
HR323	ATTACCGCTGTTCTCAAATTGTAGAAGAATGGATTGTTTCGACAGAAGCCCGTACGCTGCAGGTCGAC	Used for Clb4 tagging with 9xMyc at C-terminus

HR324	ATCCTTCCGAAACCAAAACTGAAGCAAATGGT<br><br>GTTAAGATGAGTAAGTTAATCGATGAATTCGA<br><br>GCTCG	
