# Supplementary material for: Autophagy-related protein Atg11 is essential for microtubule-mediated chromosome segregation
Source: PLoS Biol. 2025 Apr 2;23(4):e3003069. doi: 10.1371/journal.pbio.3003069 (PMC11984983; doi:10.1371/journal.pbio.3003069)
Supplement: S3 Table — (PDF) [file pbio.3003069.s011.pdf]

Supplementary Table S3: Plasmids used in this study.

S.No.	Plasmid	Construct	Source
1.	pRS313	Yeast centromere vector with a *HIS3* marker and an MCS derived from pBLUESCRIPT	Addgene Vectors
2.	pAFS125	GFP-*TUB1* expression cassette for integration at the chromosomal *URA3* locus	(90)
3.	pAKD06+CENIV	Yeast centromere vector with a *URA3* marker and another *CEN* between *GAL1* promoter and *lacZα* sequence	Prof. Santanu Kumar Ghosh, IIT-Bombay
4.	pAG32	Episomal vector with *HPH* marker	Prof. Santanu Kumar Ghosh, IIT-Bombay
5.	pUG73	Episomal vector with *LEU2* marker	Prof. Santanu Kumar Ghosh, IIT-Bombay
6.	pAW8-mCherry	Used for amplification of mCherry for gene tagging	Prof. Santanu Kumar Ghosh, IIT-Bombay
7.	pGAD-C1	Yeast two-hybrid prey vector for fusing a gene to the *GAL4* activation domain	Prof. Santanu Kumar Ghosh, IIT-Bombay
8.	pGBD-C1	Yeast two-hybrid bait vector for fusing a gene to the *GAL4* DNA-binding domain	Prof. Santanu Kumar Ghosh, IIT-Bombay
9.	pFA6A-HIS3MX6-pGAL1-VC155	Used for N-terminal tagging of proteins for bimolecular fluorescence complementation (BiFC) assay	Prof. Santanu Kumar Ghosh, IIT-Bombay
10.	pFA6A-KANMX6-pGAL1-VN173	Used for N-terminal tagging of proteins for bimolecular fluorescence complementation (BiFC) assay	Prof. Santanu Kumar Ghosh, IIT-Bombay

11.	pGADC1-*ATG11*	*ATG11* fused with the *GAL4* activation domain	This study
12.	pGBDC1-*SPC72*	*SPC72* fused with the *GAL4* DNA-binding domain	This study
13.	pGBDC1-*ATG11*	*ATG11* fused with the *GAL4* DNA-binding domain	This study
14.	pGADC1-*SPC72*	*SPC72* fused with the *GAL4* activation domain	This study
15.	pGADC1-*CNM67*	*CNM67* fused with the *GAL4* activation domain	This study
16.	pYM19	Used for tagging Clb4 with 9x-Myc	(91)
17.	pYM16	Used for tagging Pds1 with 6x-HA	(91)
18.	pTSK561	Used for tagging Atg11 with Halo tag	Addgene
