# Supplementary material for: Autophagy-related protein Atg11 is essential for microtubule-mediated chromosome segregation
Source: PLoS Biol. 2025 Apr 2;23(4):e3003069. doi: 10.1371/journal.pbio.3003069 (PMC11984983; doi:10.1371/journal.pbio.3003069)
Supplement: Attachment — Submitted filename: Response_to_Reviewers_auresp_3.pdf [file pbio.3003069.s018.pdf]

Point-by-point response to the reviewer's comments

Reza et al (2024) MS# PBIOLOGY-D-24-01398R1

Editor

Editor: The reviews are attached below. As you will see, the reviewers find the conclusions potentially interesting and significant for the field, however they also raise several concerns that would need to be addressed before we can consider the manuscript for publication. *Reviewer 1* suggests further experiments to confirm the conclusions and to strengthen the model by using live-cell microscopy, and also offers alternative interpretation of the data that should be considered. *Reviewer 2* thinks that it should be determined if Spc72 and Atg11 colocalise, and also suggests several experiments to analyse microtubule dynamics and its potential regulation by Atg11.

Authors: We thank the editor and the reviewers for finding the conclusions of our work of potential interest and significance. We have carried out live-cell microscopy, fluorescence recovery after photobleaching (FRAP), single-molecule tracking (SMT) assay, and genetic studies to address the concerns raised by *Reviewer 1*. We have now included the revised figure together with the new data from the co-localization analysis to show that Atg11 comes in proximity with Spc72. As suggested by *Reviewer 2*, we have now looked at the levels of Clb4 which regulates both aMT stability and Kar9 asymmetry in budding yeast.

Reviewer 1

R1.1: The authors explore whether autophagy-related proteins affect genome stability in budding yeast, Saccharomyces cerevisiae. First, they find that Atg11 lacking cells with poor stability of minichromosomes which suggests a segregation inaccuracies. Missegregation can arise from a variety of molecular defects and so they explore these systematically. They find that Atg11 loss leads to an arrest in metaphase, with stabilised securin. They show that Atg11 localise at spindle poles, and its association with Kar9-dependent spindle positioning. Combining these together, the work indicates changes in global microtubules (astral and kinetochore). However, the authors propose a role for changes in dynamic instability leading to segregation accuracy. The study has a variety of strengths bringing together single-molecule microscopy, genetics and live-cell analysis. The final model could be developed considering all of the data. The authors present good quality experiments, including live microscopy.

Au: We thank the reviewer for appreciating our work.

R1.2: This reviewer's main concern is that there is no direct evidence for changes in dynamic instability of kinetochore microtubules, and there is no evidence for checkpoint proteins at the kinetochore. In the absence of such direct evidence the authors can propose biorientation defects as a potential mechanism but not the mechanism for all the cell cycle changes.

Au: In the revised manuscript, we studied both the nuclear MT dynamics by fluorescence recovery after photobleaching (FRAP) assay and carried out live-cell analysis to study mono-orientation defects. An increased proportion of mono-oriented kinetochores, together with a delay of 38 s in recovery after photobleaching in *atg11Δ* cells, strongly suggests a change in nuclear microtubule (nMT) dynamics. Further, we performed additional experiments that revealed genetic interactions between *atg11Δ* and *ipl1-321* to suggest the involvement of Aurora B kinase^{Ipl1} in detecting mono-oriented kinetochores. This, in turn, activates SAC in the absence of Atg11.

R1.3: There could be alternate mechanisms of autophagy related organelle maintenance, protein degradation machineries, stress pathways which could be disrupted. Given the involvement of autophagy in mitotic catastrophe (https://doi.org/10.1038/s41598-017-14901-z), and emerging roles of mitotic autophagy (https://doi.org/10.1242/jcs.255802), Atg11 could be significant for any of these pathways. In fact, the authors see changes in a-microtubules more than KT-microtubules. So, I suggest either reinterpretation of conclusion, or exploration for direct evidence such as checkpoint protein recruitment.

Au: We concur with the reviewer that Atg11 could be involved in alternate mechanisms, considering its scaffolding function. However, we tend to believe that if indeed it were the autophagy-related alternate pathways, then the loss-of-function of Atg1, a serine/threonine kinase required for vesicle formation in autophagy and the Cvt pathway (1), where autophagy is completely abrogated, would have also shown this phenotype. That is not the case. Moreover, autophagy remains functional in *atg11Δ* cells (2). Therefore, the possibility of the autophagic degradation function of Atg11 in mediating cell cycle progression is low.

The results of FRAP, mono-orientation of chromosomes in *atg11Δ* cells, and *atg11Δ ipl1-321* genetic interactions - all point toward defects in nMT dynamics in the absence of Atg11. We have modified the model accordingly.

Major comments

R1.4: The autophagy protein screen is useful for the community. Of the various components tested, only atg11Δ cells displayed high sensitivity to TBZ but not at high temperatures - this observation could reflect a number of changes that are common to temperature change and microtubule changes (not necessarily changes in microtubule dynamics). (Figure 1)

Au: We thank the reviewer for appreciating our efforts which are going to be useful for the scientific community. *atg11Δ* cells do show sensitivity to both TBZ and a higher temperature of 37°C. TBZ sensitivity is an indication that MT-mediated processes are perturbed in *atg11Δ* cells. We concur with the reviewer that higher temperatures could lead to other defects. However, a recent paper (3), highlights that an elevated temperature alters microtubule dynamics by increasing the rate of MT depolymerization. Therefore, based on TBZ and temperature sensitivities of *atg11Δ* cells, we concluded that the loss of function of Atg11 perturbs MT-dependent processes.

We have now carried out live-cell imaging of wild-type and *atg11Δ* cells after metaphase arrest by nocodazole, followed by release to study mono-orientation defects. An increased proportion of mono-oriented kinetochores (**Figs 1A and B**), together with a delay of

38 s in recovery after photobleaching in *atg11*Δ cells (**Fig 2**), strongly suggests a change in nuclear microtubule (nMT) dynamics.

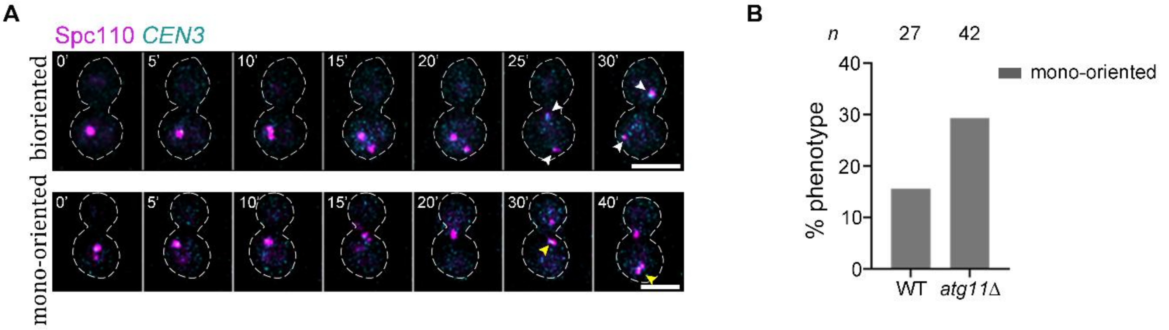

Fig 1. Biorientation defects in *atg11*Δ cells. (A) Time-lapse images of bioriented (*top*, white arrowheads) or mono-oriented (*bottom*, yellow arrowheads) kinetochores (*CEN3*-GFP) and SPBs (Spc110-mCherry) during the cell cycle in wild-type and *atg11*Δ cells after nocodazole arrest followed by release at room temperature. Scale bar, 5 μm. (B) A bar diagram representing the proportion of cells with mono-oriented kinetochores (*CEN3*-GFP) (dark gray) obtained from live-cell movies.

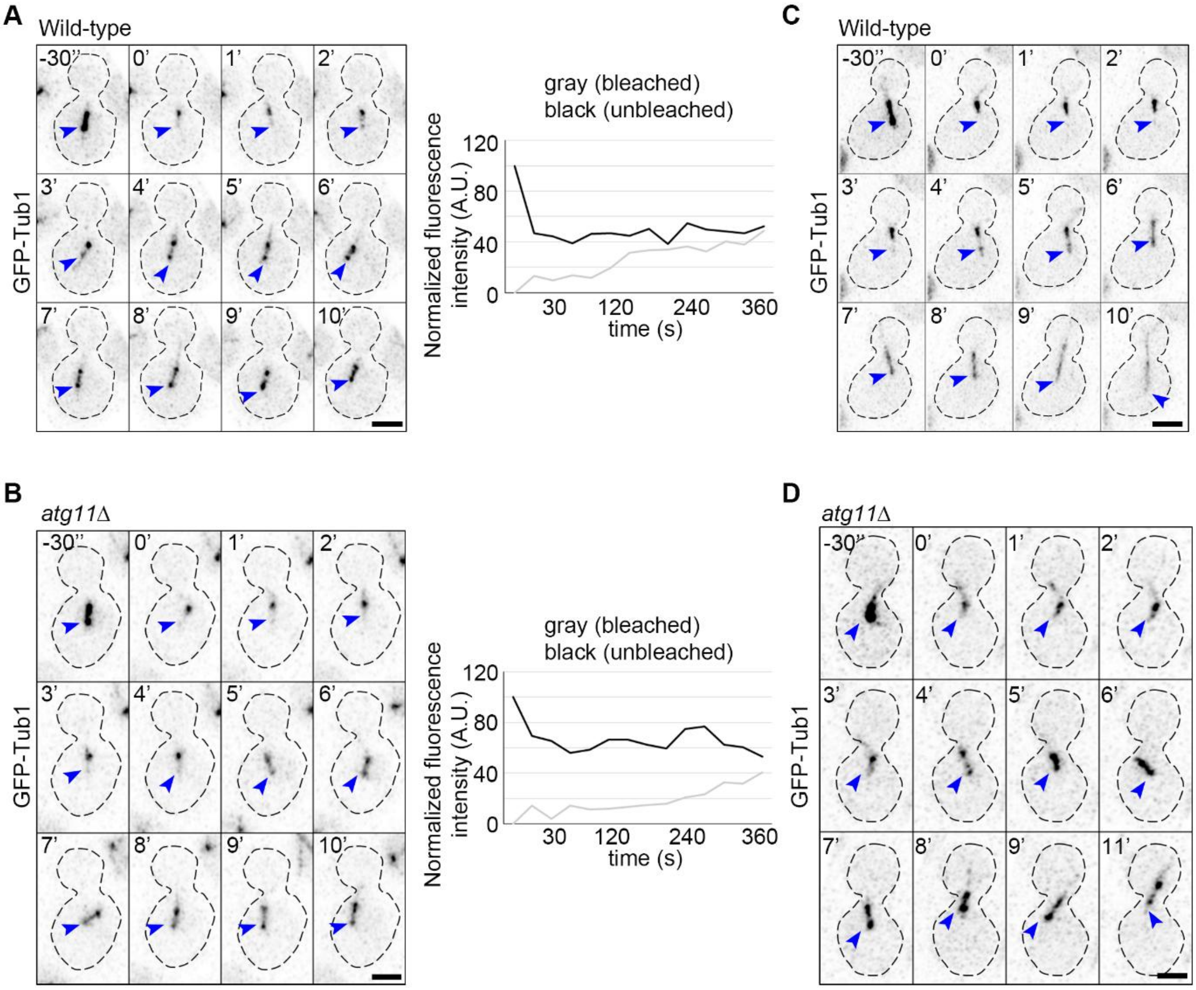

Fig 2. Fluorescence recovery after photobleaching (FRAP) of the metaphase spindle. (A-B) Time-lapse images (*left*) and graphs (*right*) representing the fluorescence recovery of photobleached metaphase spindles (blue arrowheads) in (A) wild-type and (B) *atg11*Δ cells. (C-D) Time-lapse images

(*left*) depicting the fluorescence recovery of metaphase spindle (blue arrowheads) after photobleaching and progression to anaphase in wild-type (D), and migration to the daughter bud in *atg11*Δ cells (D). t= -30 s shows the metaphase spindle before photobleaching, whereas the t= 0 time-point represents the bleached lower-half spindle. Scale bar, 2 µm. The graphs represent the quantification of fluorescence intensities of both halves (as shown) of the metaphase spindle with time.

R1.5: Deletion of *MAD2* can appear to rescue any mitotic delay or arrest and this does not directly mean that Atg11 is triggering a checkpoint. This could also be because Atg11 is affecting degradation independent of microtubule - chromosome attachment stability. (Figure 4) The authors themselves see defects in multiple phases of cell cycle (Figure 3).

Au: We thank the reviewer for raising this possibility. As mentioned in response to R1.3, if indeed it were the selective degradation which affected the cell cycle progression, then the loss-of-function of Atg1, where autophagy is completely abrogated would have also shown this phenotype. It is important to note that autophagy remains functional in *atg11*Δ cells (2). Therefore, the possibility of the autophagic degradation function of Atg11 in mediating cell cycle progression is low.

We agree with the reviewer that the *atg11*Δ cells show defects at multiple phases of the cell cycle, which is evident from the delayed Cdc14 release upon anaphase onset in these cells. We hypothesize that being a scaffolding protein, Atg11, perhaps, facilitates the transport of factors or regulators required for cell cycle progression at various stages of mitosis. However, the identification of these factors needs to be investigated.

R1.6: Significant increase in monooriented kinetochores following nocodazole release is an observation that will support the conclusion on failure to biorient (Figure 4D). This is the most significant piece of evidence to support their model and the authors could strengthen it using live-cell microscopy, and also crossing with ipl1 mutants.

Au: We thank the reviewer for the appreciation and excellent suggestions. As suggested, we have now carried out live-cell microscopy after arresting cells at metaphase using nocodazole and releasing them in fresh media. Indeed, our live-cell data supports our observation of an increased proportion of *atg11*Δ cells with mono-oriented kinetochores (**Figs 1A and B**). We have included the results in the revised manuscript (p11, lines 269-270).

We are thankful to the reviewer for suggesting a genetic cross with the *ipl1* mutant. The *atg11*Δ *ipl13-21* double mutant cells show a growth defect at 30°C and in the presence of TBZ, compared to each of the single deletion mutants. These cells display a bypass of unsegregated nuclei seen in *atg11*Δ cells with an increased proportion of segregated and often mis-segregated nuclei (**Figs 3A and B**). We have included the results in the revised manuscript (p11, lines 271-277).

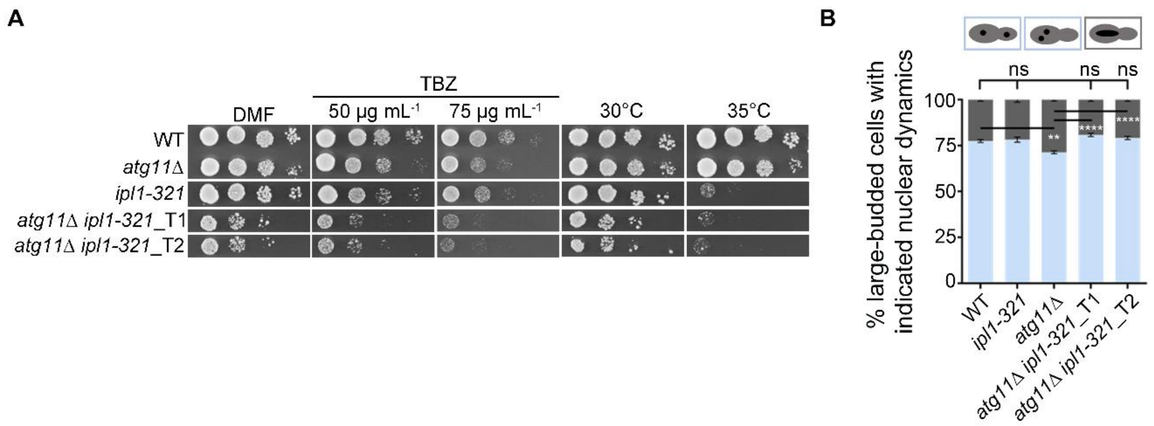

Fig 3. Ipl1-dependent activation of spindle assembly checkpoint delays cell cycle progression in *atg11*Δ cells. (A) Overnight grown cells of wild-type, single and double mutants were 10-fold serially diluted and spotted on YPD at 30°C and 35°C and in the presence of TBZ (50 and 75 µg mL^{-1}) at 30°C. Plates were photographed after 33 h of incubation. (B) A bar diagram representing the proportion of large-budded cells with stages of nuclear division in indicated strains when grown at 35°C for 6 h. Error bars show mean ± SEM. Cartoons represent nuclear morphology (black) in large-budded cells (gray). Wild-type (n>101), *atg11*Δ (n>104), *ipl1-321* (n>69), *atg11*Δ *ipl1-321*_T1 (n>101), *atg11*Δ *ipl1-321*_T2 (n>102). n, a minimum number of cells analyzed, N=3. Statistical analysis was done by two-way ANOVA using Tukey's multiple comparisons test (**p=0.0055, ****p<0.0001).

R1.7: Single molecule tracking assay could be performed in TBZ and untreated conditions/high temperature to learn about Atg11.

Au: We thank the reviewer for suggesting this experiment. We have now carried out single-molecule tracking (SMT) assay in the presence of TBZ. This analysis shows that the absence of MTs leads to a stabilization of Atg11 at the SPBs, as evidenced by a ~10-fold increase in residence time at the SPBs in the presence of TBZ (**Figs 4A and B**). The results from this analysis have been included in the revised manuscript (p8-9, lines 202-209).

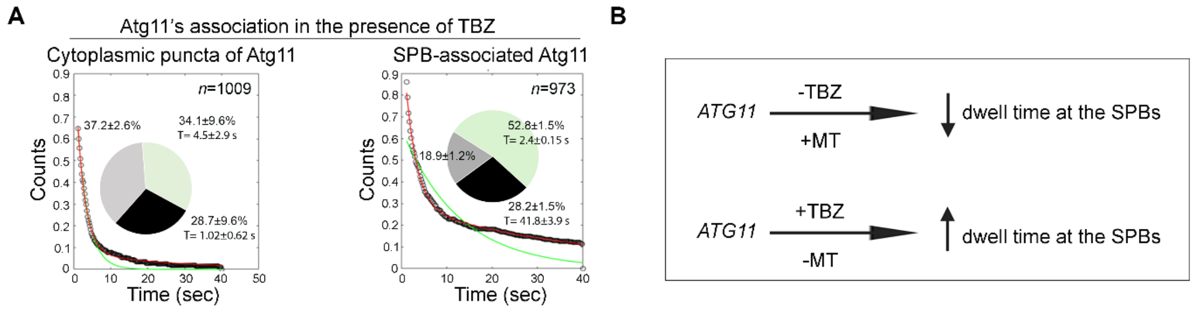

Fig 4. Single-molecule tracking in the presence of TBZ quantifies the binding dynamics of Atg11 molecules at SPBs. (A) Survival time distribution of Atg11 at cytoplasmic Atg11 puncta and SPBs was quantified from 200 ms time-interval movies in the presence of TBZ. The distribution fits well with the double exponential curve, suggesting two types of bound population for both: 1) fast fraction and 2) slow fraction. Pie charts represent the percentage of molecules unbound (gray), bound with short residence time (fast fraction, light green), and bound with long residence time (slow fraction, black). The average residence time of fast and slow fractions is presented next to their representative fractions.

n = number of tracks analyzed. (B) Schematic showing the consequence of TBZ treatment on the dynamics of Atg11 at SPBs using SMT assays.

R1.8: Figure 8, they propose a change in astral and nuclear microtubule dynamics (underpinning MT dynamics data needs to be presented). Figure 6 outcomes on MT dynamics are unclear.

Au: We have now studied the dynamics of nuclear microtubules by FRAP assays and have investigated if there are any changes in nMT dynamics upon loss of Atg11. Additionally, as suggested by the reviewer we also carried out live-cell imaging to study mono-orientation defects and genetic crossing with *atg11*Δ and *ipl1-321* mutants. Together, these results suggest an altered nMT dynamics in *atg11*Δ cells.

We would like to point out that as an outcome of altered aMT dynamics, the aMTs are shorter in *atg11*Δ cells. Asymmetric localization of Kar9 and proper integrity of aMTs are crucial for the non-random SPB inheritance. As both processes are affected in *atg11*Δ cells, we looked at SPB inheritance defects. Indeed, SPB inheritance is perturbed in *atg11*Δ cells. Therefore, in the subsequent sections, we have discussed the outcome of the changed aMT dynamics.

Minor comments

R1.9: It is unclear how atg11 localisation at spindle pole is relevant to the paper (Figure-2). It will be useful to see if Atg11 colocalises at the kinetochore or microtubule-ends.

Au: The localization of Atg11 at the SPBs is relevant due to the following reasons:

a) Our initial analysis suggested a role of Atg11 in MT-related processes and SPBs are the MT-nucleating centers. Hence, it is meaningful to know that Atg11, which is believed to be a cytoplasmic protein, is also localized to SPBs.
b) The interaction network analysis performed in this study identified Spc72 as a key interactor of Atg11. Being a component of outer plaque, it helps in nucleation of aMTs by recruiting Spc97, Spc98, and Tub4 (γ-tubulin small complex).

However, as suggested by the reviewer, we also investigated if Atg11 localizes at the kinetochores by single-molecule tracking assay. The results show no specific binding of Atg11 at the kinetochores (**Fig 5A**), suggesting that Atg11 primarily functions at SPBs in mediating MT dynamics during cell division (p11-12, lines 277-281).

A

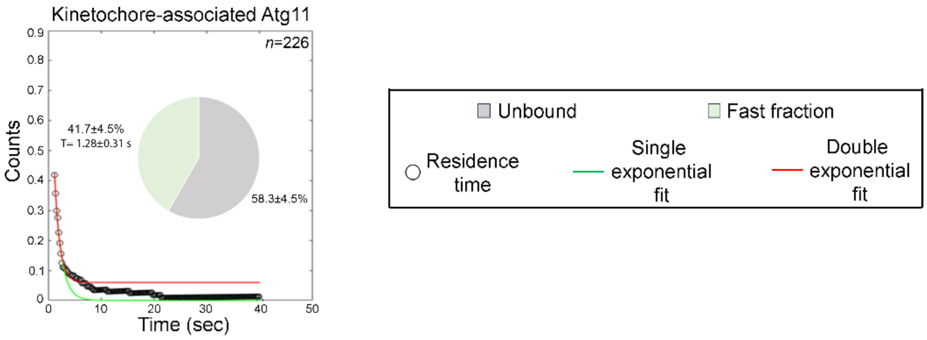

Fig 5. Single-molecule tracking of Atg11 molecules at kinetochores. (A) Survival time distribution of Atg11 at kinetochores was quantified from 200 ms time-interval movies. The distribution does not

fit well with the double exponential curve, suggesting only a fast fraction. The pie chart represents the percentage of molecules unbound (gray) and bound with short residence time (fast fraction, light green). The average residence time of fast fractions is presented next to their representative fractions. n = number of tracks analyzed.

R1.10: Experiments on Kar9 and astral microtubule changes do feel more relevant to the phenotype than failure in bioriented attachments.

Au: We concur with the reviewer that the cell cycle delay in *atg11Δ* cells translates to a delayed Cdc14 release and therefore, is not directly related to the defects in biorientation. We believe that the loss of Atg11 alters both nMT and aMT dynamics. Following the suggestion of *Reviewer 2*, we analyzed Clb4 levels and showed that the levels are stabilized in *atg11Δ* cells. Kar9 phosphorylation by Clb4/Cdc28 is known to regulate its distribution by inhibiting its loading to the mSPB (5). Western blot analysis also suggests a lower level of Clb4 at metaphase in *atg11Δ* cells. This could, in turn, facilitate the binding of Kar9 at mSPB and hence a loss of Kar9 asymmetry. It is important to note that, both Pds1 and Clb4 are the targets of the APC/C-Cdc20 complex, Pds1 is required for sister chromatid separation upon SAC inactivation and Clb4 is required for the stability of aMTs crucial for spindle positioning.

R1.11: The conclusions made in the abstract do not fully match with the interpretations made in the rest of the results (case in point figure 7).

Au: We thank the reviewer for pointing this out. We have now included the interpretation for Spc72 from Figure 7 in maintaining non-random SPB inheritance (p2, lines 46-47).

R1.12: Images require scale bars.

Au: We thank the reviewer for pointing this out. We have added scale bars to all the microscopy images included in the paper.

R1.13: Figure 4D is unclear

Au: We have now kept a schematic to represent bioriented and mono-oriented kinetochores.

Reviewer 2

R2.1: Reza et al. investigate the role of Atg11 in yeast chromosome segregation through a series of experiments. They identify Atg11 as a candidate involved in microtubule dynamics by screening for autophagy proteins affecting cell cycle progression under microtubule-depolymerizing conditions. They observe higher chromosome loss and reduced plasmid stability in Atg11-deleted strains, suggesting its role in chromosome segregation. In addition, the authors show that absence of Atg11 leads to an increased in metaphase arrested cells due to activation of the Spindle assembly checkpoint (SAC). Protein interaction analysis reveals that Atg11 interacts with Spc72. By light microscopy the observe that Atg11 transiently localizes near one of the SPB, where it might interact with Spc72. Additional live-cell imaging and single-molecule tracking confirm this interaction. Further studies show that Atg11 depletion disrupts Kar9 localization and microtubule dynamics, causing spindle

misorientation and SAC activation. Additionally, Atg11 deletion results in increased microtubule catastrophe frequency and altered rescue dynamics, further emphasizing its effect on microtubule dynamics during cell division. Based on their experiments the authors suggest that Atg11 facilitates microtubule dynamics crucial for chromosome segregation. Overall, the finding that a protein involved in autophagy affects chromosome segregation and microtubule dynamics is interesting and of relevance.

Au: We thank the reviewer for finding our study interesting and of importance to the field.

R2.2: The paper suggests that Atg11 directly affects microtubule dynamics. However, the experiments presented do not convincingly support this conclusion. It might be more plausible that Atg11 affects microtubule dynamics indirectly, possibly by regulating the degradation of cyclins or other proteins that influence the cytoskeleton.

Au: This is an important alternative explanation raised. In the revised manuscript, we have refrained from making a conclusion suggesting a direct regulation of MTs by Atg11. We also believe and propose that being a scaffolding protein, Atg11 selectively facilitates the turnover of regulators of MT at SPBs.

R2.3: The introduction of Atg11 in the manuscript is somewhat lacking, i.e. what is known, what type of protein is Atg11 etc. A more comprehensive background on Atg11's known functions and its role in cellular processes would benefit the reader. Additionally, the citations throughout this section are sparse. Providing more references to foundational and recent studies would strengthen the manuscript's scientific grounding.
Au: We have now included an introduction on Atg11 from some of the pioneering work done showing its canonical selective autophagy function (p5-6, lines 129-136). We have also included more references citing both foundational and recent studies.

R2.4: While the overall study is well-conceived, the authors should revisit some of their interpretations, particularly those that propose direct effects where indirect mechanisms might be more plausible. Additional experiments could be suggested to clarify the role of Atg11 in the observed phenomena.

Au: We have carried out additional experiments to address the suggestions of both reviewers. The revised manuscript is significantly improved as we demonstrate defects in both nuclear and astral MTs in *atg11*Δ cells.

Major comments

R2.5: The authors identify Spc72, one of the components of the spindle pole body (SPB), as a potential interactor by protein-protein interaction network analysis. Live-cell imaging (Fig 2A) reveals a transient and proximal localization of Atg11with one of the SPBs (dSPB) just prior to anaphase.
R2.5a: It would be very informative if the authors could provide a little bit more detail how the proposed co-localization of Spc72 and Atg11 was determined. Was this "quantified" by eye or was a co-localization analysis performed. The images in Figure 2A show "co-localization" at

40' and 56' but only 40' is counted as a co-localization, could the authors explain how they "discarded" 56' as not co-localizing?

Au: We would like to mention that we observed and reported a transient and proximal localization of one of the Atg11 punctum with dSPB. Using fluorescent tags, we could not detect complete co-localization between Atg11 and the SPBs by live-cell imaging. We could detect transient and partial overlap (white) of Atg11 (cyan) and Spc42 (magenta) signals. We have now modified the statement on co-localization for Fig 2A in the results section as it reads "Proximal localization was studied by a partial overlap between Atg11 (cyan) and Spc42 (magenta), giving a white-colored overlap between the two proteins" (p7, line 178-181).

As was shown in the image, the partial overlap between Atg11 and Spc42 signals was evident only at 40' and not at 56', suggesting that the two proteins are in close vicinity. Accordingly, we excluded the 56-minute time interval data in suggesting proximal localization. We, now, have included co-localization analysis for some of the time points in the revised manuscript (**Fig 6A**).

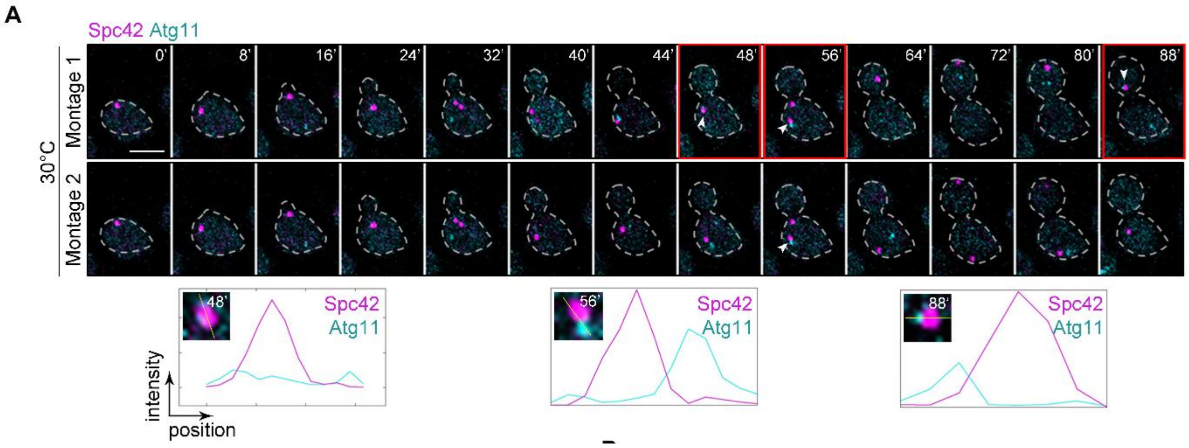

Fig 6. Localization dynamics of Atg11 with SPBs during mitosis. (A) Time-lapse images showing localization dynamics of Atg11 (sfGFP-Atg11) and SPB (Spc42-mCherry) during the cell cycle after G1 arrest followed by release at 30°C. Montage 1 and Montage 2 represent two different focal planes. The white arrowheads denote Atg11's presence proximal to the dSPB. Histogram plots represent the fluorescence intensity profile of sfGFP-Atg11 with Spc42-mCherry at 48, 56, and 88 min (marked with red boundary). Scale bar, 5 µm.

R2.5b: According to the Material and Methods the images are maximum intensity projections of z-stacks, those could be misleading in terms of co-localization as proteins might look like they are co-localizing but could indeed be in very different z-positions. Would it be possible to show co-localization in a single plane?

Au: We included maximum intensity projections of z-stacks to ensure that both the SPBs are represented in all the time frames after SPB duplication. We concur with the reviewer that maximum intensity projections could be misleading as we do not see proximal localization of Atg11 with SPBs at 40' shown previously. Therefore, as suggested by the reviewer, we have now included two panels showing both the SPBs and have included single-plane images only (**Fig 6A**). See above. We have re-analyzed all the live-cell movies which suggest that in 18 out of 24 cases, Atg11 is transiently localized proximal to the dSPB prior to anaphase onset (p7, lines 178-181).

R2.5c: Previous literature reported the localization of Atg11 as localizing to the pre-autophagosomal structure (PAS). This localization looks very similar to the localization shown here but is not discussed at all. Do the authors also observe localization to the PAS? How can this be distinguished.

Au: We thank the reviewer for raising this concern. We localized Atg11 at the vacuolar membrane by co-staining the sfGFP-Atg11 cells with CMAC and FM4-64 and have now included the PAS localization of Atg11 in the revised manuscript (**Fig 7A**) (p7, line 172-174). We asked if one of the vacuoles carrying Atg11 comes near the SPBs. To this end, we looked at the co-localization of Atg11 with a nuclear envelope marker (Nup49-mCherry) and co-stained these cells with CMAC. The analysis indeed suggests that Atg11 is at the interface of the vacuole and nuclear membrane (**Fig 7B**). This is included in the revised manuscript (p7, lines 174-175). We think that fluorescence imaging cannot distinguish between Atg11 present at PAS and SPBs. Therefore, we carried out single-molecule tracking (SMT) assays and identified two distinct populations of Atg11: the dynamic SPB-associated Atg11 and the less dynamic Atg11-punctum present in the cytosol.

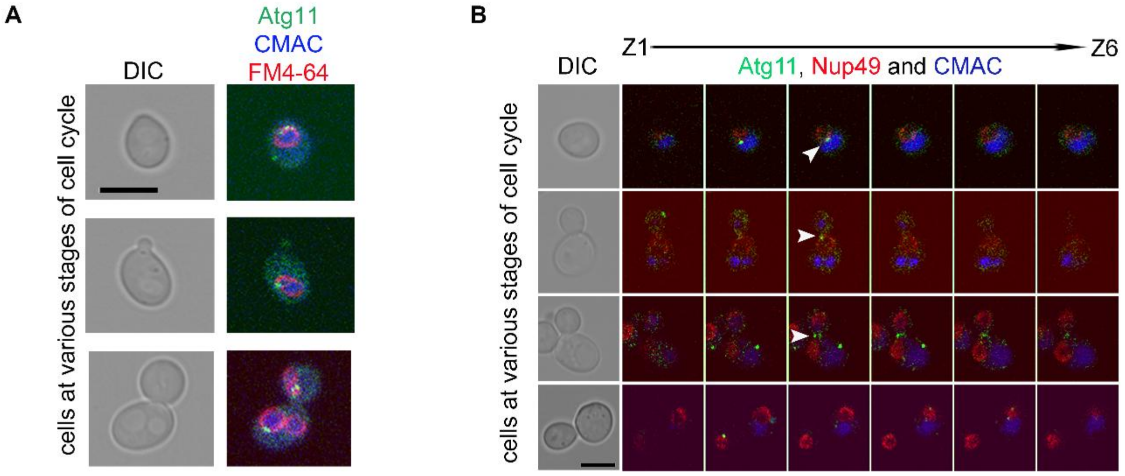

Fig 7. Cell cycle-dependent localization of Atg11. (A) Micrographs showing canonical localization of Atg11 onto the vacuolar membrane during mitosis. The sfGFP-Atg11 expressing cells were co-stained with FM4-64 (labeling vacuolar membrane, red) and with CMAC (labeling the vacuolar lumen blue). Scale bar, 5 µm. (B) Micrograph representing different focal planes showing localization of Atg11 (sfGFP-Atg11) closer to the nuclear envelope (white arrowheads), Nup49-mCherry at various cell cycle stages and co-stained with the vacuolar lumen dye CMAC. Scale bar, 6 µm.

R2.6: The authors report that Atg11 depletion leads to "more random" Kar9 localization. The authors should discuss the possible effect of erroneous Kar9 localization. I think <u>three aspects</u> are highly relevant:

R2.6a: Kar9 plays a crucial role in spindle orientation during cell division, particularly in asymmetric cell divisions. Kar9 guides the positioning of the mitotic spindle by interacting with microtubules and motor proteins, ensuring that the spindle aligns properly along the mother-bud axis in budding yeast. In addition, Kar9 was suggested to bind microtubules as well as interact with BIM1, the yeast homolog of the microtubule plus-end binding protein EB1. This could suggest that the effect on Microtubule dynamics is driven by changes in Kar9 localization

upon Atg11 depletion, rather than a direct effect of Atg11 on microtubule dynamics. This could be addressed in the discussion.

Au: We agree with the reviewer. Kar9 plays a crucial role in spindle orientation and alignment. Our results also show a two-fold increased random distribution of Kar9 which explains the two-fold increase in spindle positioning defects and a wider angle of spindle orientation upon loss of Atg11. We have discussed all three possibilities of Kar9 regulation: (a) Cdk1-Clb4-dependent, b) SAC-dependent, and c) Sumoylation, all of which help in maintaining the asymmetric distribution of Kar9 at SPBs (p18-19, lines 450-466).

R2.6b: Kar9 is regulated through a degradation pathway that starts at kinetochores. This process requires Kar9 to be near the SUMO-targeted ubiquitin ligases Slx5-Slx8, which is facilitated by transport of Kar9 into the nucleus and its recruitment to kinetochores via SUMO. If this degradation pathway is disrupted, Kar9 accumulates on cytoplasmic microtubules (cMTs), leading to spindle mislocalization into the bud. Additionally, the buildup of Kar9 on cMTs causes Bim1 to be retained in the cytoplasm, resulting in errors in chromosome segregation. This suggests that possibly the degradation of Kar9 is affected in the Atg11-depleted cells.

Au: We thank the reviewer for this suggestion. We investigated the levels of Kar9 upon cycloheximide treatment in a strain expressing Kar9 with GFP (**Fig 8A**). However, our repeated attempts could not detect any changes in Kar9 levels upon cycloheximide treatment even in wild-type, as shown previously (6). The Kar9-GFP levels seemed to follow similar dynamics both in wild-type and *atg11*Δ cells (**Fig 8B**). We are unsure if we need to change the epitope tag for Kar9 from GFP to TAP, as has been used in some previous reports (6, 7) or if cycloheximide is non-functional. We also tried using cycloheximide from a different batch and with varying concentrations, but the results were inconclusive (**Fig 8C**).

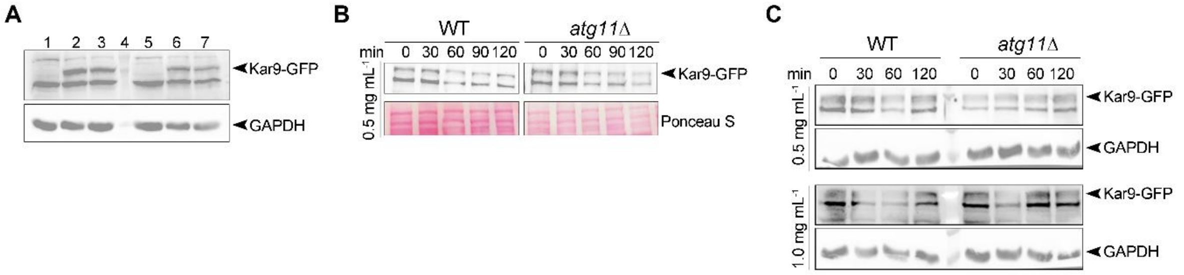

Fig 8. Kar9 levels in the presence of cycloheximide. (A) Western blot analysis shows a specific Kar9-GFP band (black arrowhead) in wild-type (lanes 2, 6) and *atg11*Δ cells (lanes 3, 7) cells expressing Kar9-GFP. Untagged control (lanes 1, 5) was used as a negative control. 20 µL protein lysates were loaded for lanes 1, 2, and 3, while 10 protein lysates were loaded for lanes 5, 6, and 7. Protein levels of GAPDH were used as a loading control. (B) Kar9 degradation dynamics (black arrowhead) in wild-type and *atg11*Δ cells in the presence of 0.5 mg mL^{-1} cycloheximide. Cells were collected at 0, 30, 60, 90, and 120 min, post cycloheximide addition, to prepare the protein samples. (C) immunoblot analysis to study Kar9 degradation dynamics in wild-type and *atg11*Δ cells in the presence of 0.5 mg mL^{-1} or 1.0 mg mL^{-1} cycloheximide. Cells were collected at 0, 30, 60, and 120 min, post cycloheximide addition, to prepare the protein samples. Protein levels of GAPDH were used as a loading control.

R2.6c: The localization and function of Kar9 are tightly regulated by phosphorylation events,

particularly by the cyclin-dependent kinase Cdc28 (CDK1 in humans). Phosphorylation affects Kar9's association with microtubules, influencing its asymmetric localization to the SPB and function during mitosis. Interestingly, both CDKs and cyclins are also targets of autophagy. Along this line, a different study by Zucca et al 2023 (PMID 36269172) found that astral microtubule (aMT) dynamics are carefully controlled. They found that aMTs stay unstable until metaphase and then stabilize at the start of anaphase. This change in aMT behavior, crucial for proper spindle orientation, depends on the degradation of the mitotic cyclin Clb4 by the Anaphase Promoting Complex (APC) with its activator subunit Cdc20 (APC/CCdc20). These findings reveal a specific role for Clb4 in regulating aMT. Is it possible that Atg11 affects the degradation of cyclins and thus Kar9 and spindles? Does the phosphorylation of Kar9 change in absence of Atg11 possibly hinting towards a role of Atg11 in degradation of cyclins? The Authors could use western blots to detect changes in phosphorylation levels of Kar9 as well as degradation of cyclins.

Au: We thank the reviewer for suggesting this. The study by Zucca et al., 2023, published in the Journal of Cell Biology, is a thorough study showing how aMTs are unstable until anaphase onset and the role of APC/C^{Cdc20}-mediated degradation of Clb4 required for aMT stability. As suggested by the reviewer, we analyzed Clb4 levels. The results suggest that the Clb4 levels are stabilized in *atg11*Δ cells (**Figs 9A and 10A**). Stabilized Clb4 levels lead to unstable aMTs. Kar9 phosphorylation by Clb4/Cdc28 is known to regulate Kar9's distribution by inhibiting its loading to the mSPB (5). It remains to be seen how a change in Clb4 levels affects Kar9 phosphorylation in the absence of Atg11 which needs to be investigated further.

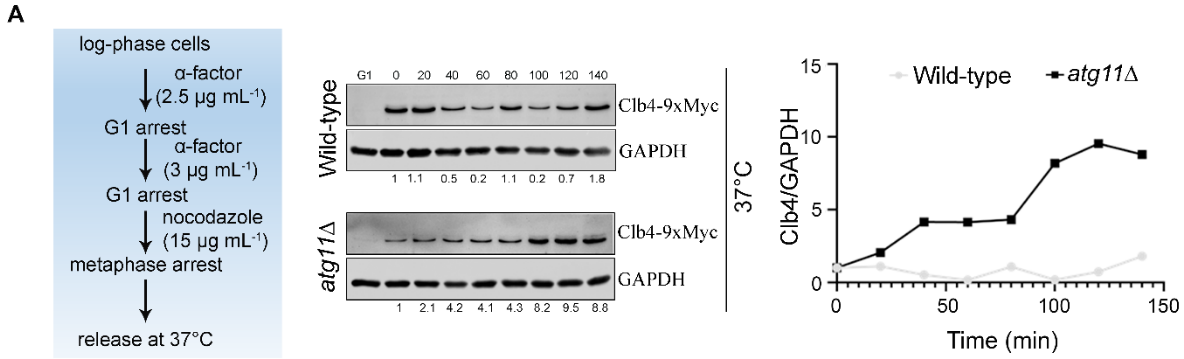

Fig 9. The *atg11*Δ cells exhibit stabilized levels of Clb4 at 37°C. (A) Schematic (*left*) showing steps involved in synchronizing followed by the release of wild-type and *atg11*Δ cells to study Clb4 protein dynamics after G1 and metaphase arrest and release at 37°C. Cells were collected every 20 min to prepare protein samples. Western blot analysis (*right*) shows the expression of Clb4-9xMyc in wild-type and *atg11*Δ cells. Protein levels of GAPDH were used as a loading control. Clb4 normalized values are indicated below each lane and the values are plotted as a line graph. Experiments were repeated twice with similar dynamics.

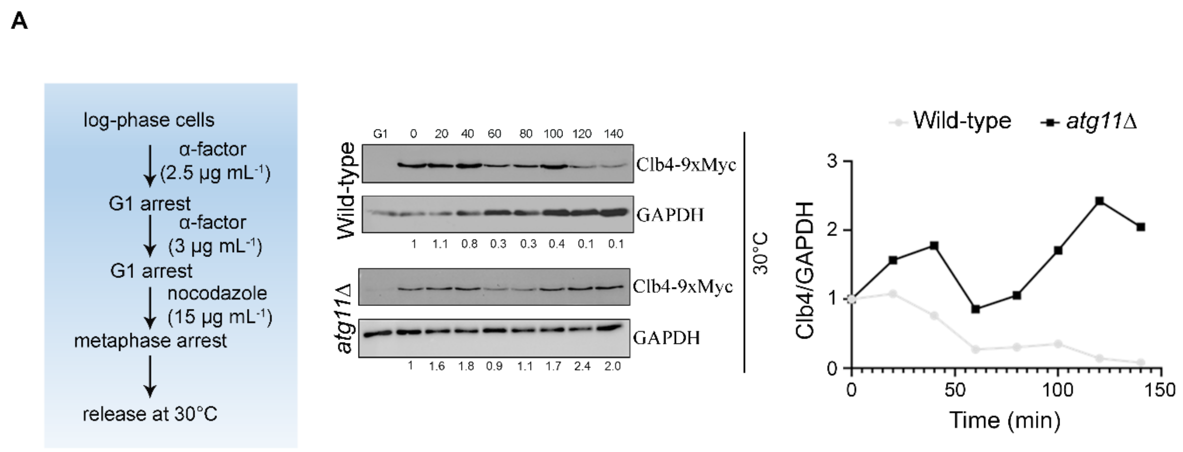

Fig 10. The *atg11*Δ cells exhibit stabilized levels of Clb4 at 30°C. (A) Schematic (*left*) showing steps involved in synchronizing followed by the release of wild-type and *atg11*Δ cells to study Clb4 protein dynamics after G1 and metaphase arrest and release at 30°C. Cells were collected every 20 min to prepare protein samples. Western blot analysis (*right*) shows the expression of Clb4-9xMyc in wild-type and *atg11*Δ cells. Protein levels of GAPDH were used as a loading control. Clb4 normalized values are indicated below each lane and the values are plotted as a line graph. The experiments were repeated twice with similar dynamics.

R2.7: Are microtubule dynamics changed globally in the entire cells or locally (SPB specific)? Or rather, given that Atg11 localization is asymmetric, are microtubule dynamics on the SPB with Atg11 different from the one without? Microtubule dynamics could be quantified locally.

Au: We would like to mention that the astral microtubule (aMT) dynamics were studied only from the old SPB (dSPB) which nucleates longer MTs. The aMTs from the other SPB (mSPB) at the pre-anaphase stage in most of the cells were shorter and difficult to quantify for all the time points. We have mentioned this in the revised manuscript in the materials and methods section (p33, lines 803-804).
In the revised manuscript, we have also carried out live-cell imaging of wild-type and *atg11*Δ cells after metaphase arrest by nocodazole, followed by release to study bi-orientation defects. An increased proportion of mono-oriented kinetochores (**Figs 11A and B**), together with a delay of 38 s in recovery after photobleaching in *atg11*Δ cells (**Fig 12**), strongly suggests a change in nuclear microtubule (nMT) dynamics.

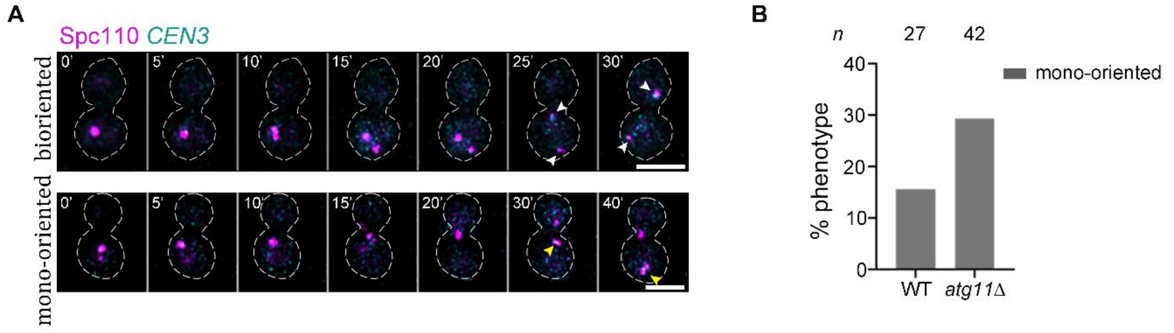

Fig 11. Biorientation defects in *atg11*Δ cells. (A) Time-lapse images of bioriented (*top*, white arrowheads) or mono-oriented (*bottom*, yellow arrowheads) kinetochores (*CEN3*-GFP) and SPBs

(Spc110-mCherry) during the cell cycle in wild-type and *atg11*Δ cells after nocodazole arrest followed by release at room temperature. Scale bar, 5 µm. (B) A bar diagram representing the proportion of cells with mono-oriented kinetochores (*CEN3*-GFP) (dark gray) obtained from live-cell movies.

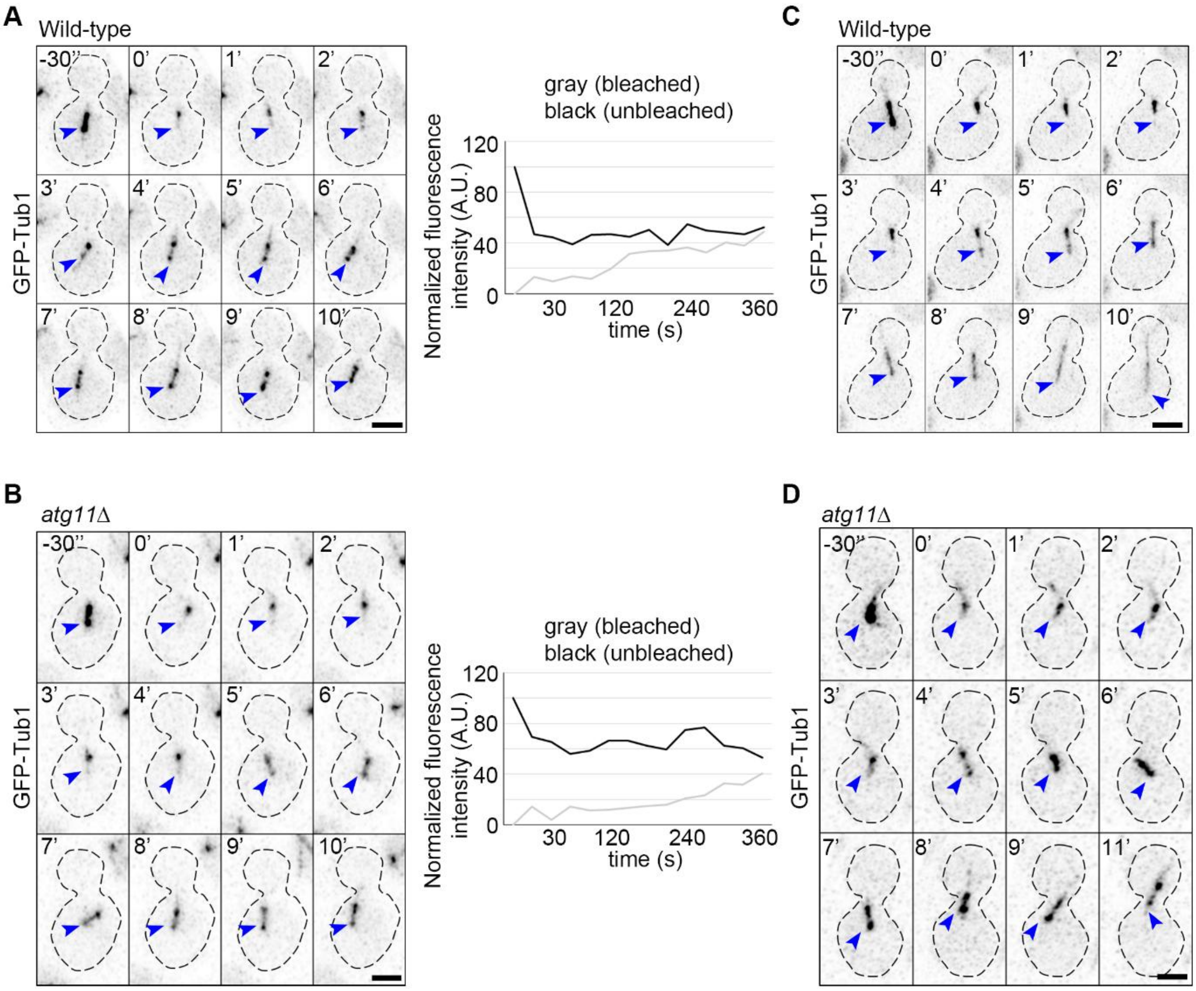

Fig 12. Fluorescence recovery after photobleaching (FRAP) of the metaphase spindle. (A-B) Time-lapse images (*left*) and graphs (*right*) representing the fluorescence recovery of photobleached metaphase spindles (blue arrowheads) in (A) wild-type and (B) *atg11*Δ cells. (C-D) Time-lapse images (*left*) depicting the fluorescence recovery of metaphase spindle (blue arrowheads) after photobleaching and progression to anaphase in wild-type (D), and migration to the daughter bud in *atg11*Δ cells (D). t= -30 s shows the metaphase spindle before photobleaching, whereas the t= 0 time-point represents the bleached lower-half spindle. Scale bar, 2 µm. The graphs represent the quantification of fluorescence intensities of both halves (as shown) of the metaphase spindle with time.

R2.8: Based on their experiments the authors suggest that "Atg11 facilitates microtubule dynamics crucial for chromosome segregation". The data clearly shows that cells depleted of Atg11 have altered microtubule dynamics, however the data does not allow the conclusion that Atg11 directly regulates microtubule dynamics. The discussion of the data should reflect this.

Au: We concur with the reviewer that our results do not suggest Atg11-mediated direct regulation of MT dynamics and we have modified our discussion and included some of the possibilities in the discussion to reflect this.

Minor comments

R2.9: Figure 7B label for atg11 depleted is missing.

Au: Figure 7B shows all kinds of possibilities for the asymmetric distribution of Kar9 and Spc72 in wild-type cells. Some percentage of cells display this distribution even in the wild-type, which increases upon loss-of-function of proteins which regulate the asymmetric distribution of either Kar9 or Spc72 or both. This is based on representation depicted in a couple of studies (8, 9). Therefore, we had not included *atg11Δ* in Figure 7B.

R2.10: Those two lines contradict each other in terms of effects on rescue frequency. A significant increase in rescue events should also lead to an increase in rescue frequency. However, authors here report no difference in rescue frequency: p28, line 477: " The atg11Δ cells displayed a significantly increased catastrophe frequency (Fig. 6C), while the rescue frequencies were comparable to those of the wild-type" p29, line 493:" The atg11Δ cells also showed a significant increase in the number of rescue events".

Au: Rescue frequency was calculated as the total number of depolymerization-to-polymerization transitions divided by the total time of all shrinkage events and minimum length at rescue. While the rescue events represent the total number of rescue events per aMT in a cell. Therefore, even though the number of rescue events was more in *atg11Δ* cells, the rescue frequency is insignificant because the total number of events is divided by the time and length of aMTs. The introduction of two factors makes rescue frequency non-significant between wild-type and *atg11Δ* cells. Also, we would like to mention that the units for supporting figures S5A, C, D, and F in the previous draft were overlooked and have been changed in the revised manuscript.

References

1. Kim J, Kamada Y, Stromhaug PE, Guan J, Hefner-Gravink A, Baba M, et al. Cvt9/Gsa9 functions in sequestering selective cytosolic cargo destined for the vacuole. The Journal of cell biology. 2001;153(2):381-96.
2. Cheong H, Yorimitsu T, Reggiori F, Legakis JE, Wang CW, Klionsky DJ. Atg17 regulates the magnitude of the autophagic response. Mol Biol Cell. 2005;16(7):3438-53.
3. Li G, Moore JK. Microtubule dynamics at low temperature: evidence that tubulin recycling limits assembly. Mol Biol Cell. 2020;31(11):1154-66.
4. Doheny KF, Sorger PK, Hyman AA, Tugendreich S, Spencer F, Hieter P. Identification of essential components of the *S. cerevisiae* kinetochore. Cell. 1993;73(4):761-74.
5. Liakopoulos D, Kusch J, Grava S, Vogel J, Barral Y. Asymmetric loading of Kar9 onto spindle poles and microtubules ensures proper spindle alignment. Cell. 2003;112(4):561-74.
6. Schweiggert J, Stevermann L, Panigada D, Kammerer D, Liakopoulos D. Regulation of a spindle positioning factor at kinetochores by SUMO-targeted ubiquitin ligases. Developmental Cell. 2016;36(4):415-27.

7. Leisner C, Kammerer D, Denoth A, Britschi M, Barral Y, Liakopoulos D. Regulation of mitotic spindle asymmetry by SUMO and the spindle-assembly checkpoint in yeast. Current Biology. 2008;18(16):1249-55.
8. Manzano-Lopez J, Matellan L, Alvarez-Llamas A, Blanco-Mira JC, Monje-Casas F. Asymmetric inheritance of spindle microtubule-organizing centres preserves replicative lifespan. Nat Cell Biol. 2019;21(8):952-65.
9. Matellan L, Manzano-Lopez J, Monje-Casas F. Polo-like kinase acts as a molecular timer that safeguards the asymmetric fate of spindle microtubule-organizing centers. Elife. 2020;9.
