# Supplementary material for: Autophagy-related protein Atg11 is essential for microtubule-mediated chromosome segregation
Source: PLoS Biol. 2025 Apr 2;23(4):e3003069. doi: 10.1371/journal.pbio.3003069 (PMC11984983; doi:10.1371/journal.pbio.3003069)
Supplement: Attachment — Submitted filename: Review_RezaETAL.docx [file pbio.3003069.s015.docx]

Reza et al. investigate the role of Atg11 in yeast chromosome segregation through a series of experiments. They identify Atg11 as a candidate involved in microtubule dynamics by screening for autophagy proteins affecting cell cycle progression under microtubule-depolymerizing conditions. They observe higher chromosome loss and reduced plasmid stability in Atg11-deleted strains, suggesting its role in chromosome segregation. In addition, the authors show that absence of Atg11 leads to an increased in metaphase arrested cells due to activation of the Spindle assembly checkpoint (SAC). Protein interaction analysis reveals that Atg11 interacts with Spc72. By light microscopy the observe that Atg11 transiently localizes near one of the SPB, where it might interact with Spc72. Additional live-cell imaging and single-molecule tracking confirm this interaction. Further studies show that Atg11 depletion disrupts Kar9 localization and microtubule dynamics, causing spindle misorientation and SAC activation. Additionally, Atg11 deletion results in increased microtubule catastrophe frequency and altered rescue dynamics, further emphasizing its effect on microtubule dynamics during cell division. Based on their experiments the authors suggest that Atg11 facilitates microtubule dynamics crucial for chromosome segregation.

Overall, the finding that a protein involved in autophagy affects chromosome segregation and microtubule dynamics is interesting and of relevance. The paper suggests that Atg11 directly affects microtubule dynamics. However, the experiments presented do not convincingly support this conclusion. It might be more plausible that Atg11 affects microtubule dynamics indirectly, possibly by regulating the degradation of cyclins or other proteins that influence the cytoskeleton. The introduction of Atg11 in the manuscript is somewhat lacking, i.e. what is known, what type of protein is Atg11 etc. A more comprehensive background on Atg11's known functions and its role in cellular processes would benefit the reader. Additionally, the citations throughout this section are sparse. Providing more references to foundational and recent studies would strengthen the manuscript's scientific grounding. While the overall study is well-conceived, the authors should revisit some of their interpretations, particularly those that propose direct effects where indirect mechanisms might be more plausible. Additional experiments could be suggested to clarify the role of Atg11 in the observed phenomena.

Major comments:

1) The authors Identify Spc72, one of the components of the spindle pole body (SPB), as a potential interactor by protein-protein interaction network analysis. Live-cell imaging (Fig 2A) reveals a transient and proximal localization of Atg11with on of the SPBs (dSPB) just prior to anaphase.

a) It would be very informative if the authors could provide a little bit more detail how the proposed co-localization of Spc72 and Atg11 was determined. Was this "quantified" by eye or was a co-localization analysis performed. The images in Figure 2A show "co-localization" at 40' and 56' but only 40' is counted as a co-localization, could the authors explain how they "discarded" 56' as not co-localizing?

b) According to the Material and Methods the images are maximum intensity projections of z-stacks, those could be misleading in terms of co-localization as proteins might look like they are co-localizing but could indeed be in very different z-positions. Would it be possible to show co-localization in a single plane?

c) Previous literature reported the localization of Atg11 as localizing to the pre-autophagosomal structure (PAS). This localization looks very similar to the localization shown here but is not discussed at all. Do the authors also observe localization to the PAS? How can this be distinguished.

2) The authors report that Atg11 depletion leads to "more random" Kar9 localization. The authors should discuss the possible effect of erroneous Kar9 localization. I think three aspects are highly relevant:

a) Kar9 plays a crucial role in spindle orientation during cell division, particularly in asymmetric cell divisions. Kar9 guides the positioning of the mitotic spindle by interacting with microtubules and motor proteins, ensuring that the spindle aligns properly along the mother-bud axis in budding yeast. In addition, Kar9 was suggested to bind microtubules as well as interact with BIM1, the yeast homolog of the microtubule plus-end binding protein EB1. This could suggest that the effect on Microtubule dynamics is driven by changes in Kar9 localization upon Atg11 depletion, rather than a direct effect of Atg11 on microtubule dynamics. This could be addressed in the discussion.

b) Kar9 is regulated through a degradation pathway that starts at kinetochores. This process requires Kar9 to be near the SUMO-targeted ubiquitin ligases Slx5-Slx8, which is facilitated by a transport of Kar9 into the nucleus and its recruitment to kinetochores via SUMO. If this degradation pathway is disrupted, Kar9 accumulates on cytoplasmic microtubules (cMTs), leading to spindle mislocalization into the bud. Additionally, the buildup of Kar9 on cMTs causes Bim1 to be retained in the cytoplasm, resulting in errors in chromosome segregation.

This suggests that possibly the degradation of Kar9 could be affected in the Atg11 depleted cells.

c) The localization and function of Kar9 are tightly regulated by phosphorylation events, particularly by the cyclin-dependent kinase Cdc28 (CDK1 in humans). Phosphorylation affects Kar9’s association with microtubules, influencing its asymmetric localization to the SPB and function during mitosis. Interestingly, both CDKs and cyclins are also targets of autophagy.

Along this line, a different study by Zucca et al 2023 (PMID 36269172) found that astral microtubule (aMT) dynamics are carefully controlled. They found that aMTs stay unstable until metaphase and then stabilize at the start of anaphase. This change in aMT behavior, crucial for proper spindle orientation, depends on the degradation of the mitotic cyclin Clb4 by the Anaphase Promoting Complex (APC) with its activator subunit Cdc20 (APC/CCdc20). These findings reveal a specific role for Clb4 in regulating aMT.

Is it possible that Atg11 affects the degradation of cyclins and thus Kar9 and spindles? Does the phosphorylation of Kar9 change in absence of Atg11 possibly hinting towards a role of Atg11 in degradation of cyclins?

The Authors could use western blots to detect changes in phosporylation levels of Kar9 as well as degradation of cyclins.

3) Are microtubule dynamics changed globally in the entire cells or locally (SPB specific)? Or rather, given that Atg11 localization is asymmetric, are microtubule dynamics on the SPB with Atg11 different from the one without?

Microtubule dynamics could be quantified locally.

4) Based on their experiments the authors suggest that "Atg11 facilitates microtubule dynamics crucial for chromosome segregation". The data clearly shows that cells depleted of Atg11 have altered microtubule dynamics, however the data does not allow the conclusion that Atg11 directly regulates microtubule dynamics. The discussion of the data should reflect this.

Minor Comments:

Figure 7B label for atg11 depleted is missing

Those two lines contradict each other in terms of effects on rescue frequency. A significant increase in rescue events should also lead to an increase in rescue frequency. However, authors here report no difference in rescue frequency:

p28, line 477: " *The atg11Δ cells displayed a significantly increased catastrophe frequency (Fig. 6C), while the rescue frequencies were comparable to those of the wild-type"*

p29, line 493:" *The atg11Δ cells also showed a significant increase in the number of rescue events"*
